# The Melanocortin System behind the Dysfunctional Eating Behaviors

**DOI:** 10.3390/nu12113502

**Published:** 2020-11-14

**Authors:** Emanuela Micioni Di Bonaventura, Luca Botticelli, Daniele Tomassoni, Seyed Khosrow Tayebati, Maria Vittoria Micioni Di Bonaventura, Carlo Cifani

**Affiliations:** 1 School of Pharmacy, University of Camerino, 62032 Camerino, Italy; emanuela.micioni@unicam.it (E.M.D.B.); luca.botticelli@unicam.it (L.B.); khosrow.tayebati@unicam.it (S.K.T.); carlo.cifani@unicam.it (C.C.); 2 School of Bioscience and Veterinary Medicine, University of Camerino, 62032 Camerino, Italy; daniele.tomassoni@unicam.it

**Keywords:** melanocortin system, MC3R, MC4R, eating disorders, binge eating disorder, food reward, obesity, *MC4R* mutation, rs17782313, stress

## Abstract

The dysfunction of melanocortin signaling has been associated with obesity, given the important role in the regulation of energy homeostasis, food intake, satiety and body weight. In the hypothalamus, the melanocortin-3 receptor (MC3R) and melanocortin-4 receptor (MC4R) contribute to the stability of these processes, but MC3R and MC4R are also localized in the mesolimbic dopamine system, the region that responds to the reinforcing properties of highly palatable food (HPF) and where these two receptors seem to affect food reward and motivation. Loss of function of the MC4R, resulting from genetic mutations, leads to overeating in humans, but to date, a clear understanding of the underlying mechanisms and behaviors that promote overconsumption of caloric foods remains unknown. Moreover, the MC4R demonstrated to be a crucial modulator of the stress response, factor that is known to be strictly related to binge eating behavior. In this review, we will explore the preclinical and clinical studies, and the controversies regarding the involvement of melanocortin system in altered eating patterns, especially binge eating behavior, food reward and motivation.

## 1. Introduction

Nowadays, the increased consumption of food highly rich in fat, sugar and palatable components has fueled the so called Western diet, leading to excessive and non-homeostatic feeding behavior that impacts the quality of life [1,2]. The melanocortin system, known to be a key pathway in the regulation of food intake, body weight and energy balance [3,4,5,6], has been proposed as a possible underlying factor not only in obesity, in which there is evidence of a consistent relationship [7,8,9,10,11,12,13,14], but also in several dysfunctional eating patterns [15,16,17,18,19,20] that can lead to obesity, modulating the motivation for hedonic properties of food [21,22,23]. Among the altered feeding patterns, binge eating behavior is one of the most studied, due to the overlaps that exist with obesity [24,25,26], and melanocortin signaling can influence reward-related behaviors, given the presence of melanocortin receptors (MCRs) not only in the hypothalamus, but also in reward-related brain areas such as in the mesolimbic dopamine pathway [27,28].

Binge eating is a typical feature in eating disorders, in particular Bulimia Nervosa, binge/purging subtype of Anorexia Nervosa and Binge Eating Disorder (BED). A binge eating episode is characterized by an unusual consumption of a large amount of food that most people would not eat in the same discrete period of time, connected with the inability to stop overeating, accompanied by feelings of guilt, shame and regret [29]. Differently from Bulimia Nervosa and Anorexia Nervosa, BED is characterized by recurrent episodes of binge eating not followed by inappropriate compensatory behaviors, such as vomiting, prolonged fasting, or excessive exercise for controlling weight gain [29]. BED is the most prevalent eating disorder in adolescents and young women [24,25,30], and it is associated in some instances with overweight or obesity [31,32]. The subgroup of obese individuals that suffers also from BED seems to increase food-related impulsivity and reward sensitivity in comparison to obese people without BED [33,34]. Additionally, food craving is significantly higher under negative emotional states (including disappointment, anger, guilt, depressive symptoms) [35,36] and stress exposure [37,38,39] in obese binge eaters rather than obese. Thus, binge eating is a risk factor for obesity and, at the same time, overweight and obesity might enhance the possibility to engage binge eating behavior [40]. In light of these interconnected aberrant feeding patterns and the involvement of the MCRs in overeating and stress, the aim of this review is to revise the current literature on PubMed, regarding the role of the melanocortin system as a mutual underlying factor that may increase the susceptibility to develop aberrant eating behaviors. After a brief summary of the localization and the physiological functions of the melanocortin system, we will describe the role of melanocortin-3 receptor (MC3R) and melanocortin-4 receptor (MC4R) on food intake, focusing on their interaction with the brain reward system. Subsequently, we will highlight the impact of genetic mutations of MC4R on food consumption in humans. Finally, the melanocortin system, principally via MC4R, will be explored in stress response, considering stress as a key factor triggering altered feeding patterns.

## 2. An Overview of the Melanocortin Receptors in the Control of Food Intake

Pro-opiomelanocortin (POMC) is the precursor molecule of α-melanocyte-stimulating hormone (α-MSH), one of its proteolytic cleavage products, which has a regulatory role in feeding related behavior and satiety; the other active peptides are β-MSH, γ-MSH, adrenocorticotropic hormone (ACTH) and β-endorphin [3,4,9]. Localization of POMC neurons in the central nervous system (CNS) is in the arcuate nucleus of the hypothalamus (ARC) and in the nucleus of the tractus solitarius (NTS) of the brainstem, areas implicated in body weight loss, energy homeostasis and signaling of satiety, showing anorexigenic effects [4]. Adjacent to POMC cells, in the hypothalamic ARC, are localized agouti-related protein (AgRP) neurons and the neuropeptide Y (NPY) neurons producing, respectively, the endogenous antagonist of MCRs AgRP and the orexigenic neuropeptide NPY, both able to increase food intake [3,41,42]. In the 1990s, the first MCRs were initially cloned, and, subsequently, all five MCRs, members of the superfamily of G protein-coupled receptors, have been identified [43,44,45,46]. MC3R and MC4R are widely expressed in CNS, and, binding the endogenous MCRs agonist, α-MSH, are able to activate adenylate cyclase to elevate intracellular cAMP levels, generating an anorexigenic signal [41,47], regulating the homeostasis of energy intake and feeding behavior and suppressing food consumption [4,6,16]. Conversely, MC1R, MC2R and MC5R are primarily found in the periphery: the MC1R especially in the melanocytes, the MC2R in the adrenal cortex and MC5R in the exocrine glands [4,48].

The MC3R is predominantly expressed in the brain within the hypothalamus, mainly in the ARC and less in the dorsomedial portion of the ventromedial nucleus, anteroventral preoptic area, posterior hypothalamic area, the medial preoptic area and paraventricular nucleus (PVN) of the hypothalamus, but there is evidence of MC3R moderately localized also in the limbic system, in ventral tegmental area (VTA), central linear nucleus of raphe, in the lateral nucleus of the septum and in the medial habenula nucleus of the thalamus [43,46,48,49].

In contrast with MC3R, the MC4R has a more widespread expression in the CNS; indeed, the MC4R shows high prevalence in hypothalamic sites including PVN, the medial preoptic area, anterior hypothalamic nucleus, ventromedial nucleus of the hypothalamus, dorsomedial nucleus of the hypothalamus, tuberomammillary nucleus and other several hypothalamic areas, but it is also strongly expressed in the brainstem and moderately in the cortex, hippocampus, corpus striatum, amygdala, thalamus, spinal cord and also detected in the peripheral nervous system [27,43,48,50,51].

In the brain, the distinct localization, more widely for MC4R than for MC3R, also reflects a different binding with the peptides deriving from POMC cleavage: α-MSH and γ-MSH have high affinity for MC3R; meanwhile, MC4R is preferentially bound by α-MSH and less by γ-MSH [44,46,50,51]. Moreover, AgRP, endogenous antagonist of MCRs, has high affinity for both these receptors [41,42], reflecting a differential regulation of the metabolic response and food consumption [7,42,52]. Furthermore, in the hypothalamus, the melanocortin pathway interacts with other crucial hormones, such as leptin and insulin, which promote the processing of POMC to the anorexigenic α-MSH, signaling a decreased energy intake and contributing to the fed state (for details see ref. [12,53,54,55]). In addition, another functional interaction of the melanocortin system in the ARC nucleus is with the orexigenic neuropeptide Nociceptin/Orphanin FQ [56,57], which exerts an inhibitory influence on α-MSH cells [58], and is strictly involved in stress mechanisms [59,60] and binge eating behavior [56,61,62,63,64].

Preliminary information about the functions and physiological role related to feeding of MC3R and MC4R was provided by studies with the deletion of these MCRs in mice, which developed obesity, increased adipose mass, hyperphagia and lack of appetite control, in particular more pronounced in MC4R knockout (KO) mice rather than MC3R KO mice, even though mice lacking both receptors become significantly heavier than MC4R KO [11,16,65,66,67,68]. Additionally, all the previous effects, characteristic of severe obesity, are predominantly linked to *MC4R* mutations and defects in MC4R signaling in humans, compared to the alterations of MC3R, which frequently cause only moderate obesity or limited hyperphagia; to date, the role of MC3R remains an element that needs to be clarified [11,13,14,69,70,71,72]. Taking into account all these findings, it is interesting to explore the studies conducted so far regarding the association of MCRs with compulsive eating, food reward and motivation, and to support the possibility of their implication in binge eating behavior.

## 3. Melanocortin Receptors in Feeding

### 3.1. MC3R

#### 3.1.1. MC3R: Preclinical Studies on Eating Behavior

The MC3R, compared to the MC4R subtype, exhibits a more limited distribution in the brain, being predominantly found in the hypothalamic nuclei and limbic regions, with dense expression in the ARC, ventromedial hypothalamus, VTA and medial habenula, structures in which it is supposed to regulate energy homeostasis and food seeking behavior [46,50,73,74,75]. MC3R and MC4R KO mice have been used to investigate the role of each receptor in regulating energy homeostasis, and many studies revealed that MC3Rs and MC4Rs might function independently, playing a complementary but non-redundant role in the regulation of energy balance [65,66,68,76]. Targeted deletion of the MC3R gene in mice promotes a modest obesity syndrome and increased accumulation of fat mass that is not related to hyperphagia, with a normal anorectic response to melanocortin agonists [65,66], suggesting that this receptor could be mostly involved in the regulation of energy homeostasis and metabolic processes, rather than in the control of feeding behavior. However, a study by Zhang et al. showed that MC3Rs and MC4Rs are of approximately equal importance in preventing weight gain during a high-fat chow diet, and that the absence of MC3Rs compromises leptin’s ability to decrease food consumption [76], evidencing an altered anorectic response in MC3R null mice. Moreover, male MC3R KO mice, backcrossed onto the C57BL/6J background, showed a mild hyperphagia after exposure to a purified high-fat diet [6]. Sutton et al. demonstrated that obesity associated with MC3R deficiency is dependent on the dietary fat, considering that, if exposed to a low-fat diet, MC3R KO mice exhibited a modest increase in adiposity and a normal body weight, while during a high-fat diet, fat mass was comparable to that of MC4R KO littermates [68]. Additionally, MC3R KO mice were not hyperphagic under a low-fat diet, but showed a modest increase in food consumption under the high-fat diet, an effect that was gender specific, being mainly observed in male mice [68]. A recent experiment in mice with “humanized” MC3Rs further evidenced the role of the MC3R in appetite control: in this mouse model, the murine MC3R was replaced with the Wild Type (WT) human MC3R (MC3R^hWT/hWT^) or the double-mutant C17A (Thr6Lys) + G241A (Val81Ile) human MC3R (MC3R^hDM/hDM^) [77], characterized by a reduced receptor binding, signaling transduction and less protein expression, and associated with a greater risk of childhood obesity in human homozygous carriers [78,79,80]. Mutant homozygous mice with the double mutation (MC3R^hDM/hDM^) had an increased adiposity and energy intake, compared to WT human MC3R (MC3R^hWT/hWT^) littermates and were also hyperphagic [77], highlighting the contribution of MC3R signaling to energy homeostasis, metabolism and feeding behavior.

The behavioral phenotype linked to MC3R-deficiency may be also contextual and dependent on energy balance. In fact, MC3R-deficient mice appear to be less sensitive to the “pain” of hunger, and are not motivated to avoid unpleasant experiences associated with nutrient scarcity [81], as described by investigations using hypocaloric restricting feeding protocols. In this context, MC3Rs seem to be essential for entrainment of anticipatory behavior toward feeding time [81,82]. Food anticipatory behavior, consisting of a progressive rise of activity preceding food presentation, assessed using running wheels and measuring home cage activity, is attenuated in MC3R KO mice, compared to WT, under a restricting feeding protocol [73,82,83]. Moreover, the same mice did not exhibit the increased wakefulness generally coincident with food presentation and normally observed in non-mutant rodents [82]. Hypocaloric feeding protocols are known to promote binge-like eating behavior in WT mice, which reduce meal frequency but increase meal size and duration, with most of the food consumed within the first hour of presentation [21,83,84]. This behavioral phenotype is markedly attenuated in MC3R KO mice, without compensation in the feeding cycle later or changes in the meal structure, a finding that supports the essential role of an intact MC3R signaling in the compulsive eating response, observed after exposure to situations of poor nutrient availability and prolonged negative energy balance [83,84]. Additionally, the motivation to self-administer a food reward is markedly attenuated in MC3R-deficient mice, exposed to a caloric restriction protocol, while being normal if mice are fed in ad libitum conditions, reducing self-administration of chocolate flavored pellets [21]. The abnormal behavioral features associated with the deletion of MC3Rs could be partially explained by the neuroendocrine alterations found in the brain of MC3R-deficient mice, which failed to present the increase in the potent orexigenic neuropeptides AgRP and NPY during fasting and hypocaloric conditions [5,84,85]. Intriguingly, MC3R-deficient mice also exhibit altered responses of the hypothalamic-pituitary-adrenal (HPA) axis during caloric restriction, showing a lack of corticosterone serum increase in response to fasting, which instead is found in WT mice [84,85]. Furthermore, the dysregulation of fasting-induced corticosterone release was accompanied by a defect in the upregulation of hypothalamic corticotropin-releasing hormone (CRH) mRNA in the MC3R KO mice [85], indicating that both hypothalamic and adrenal functions are compromised by the absence of this receptor. Considering both the dysfunctional eating behaviors and the altered activity of the HPA axis observed in MC3R KO mice during fasting, future studies should be conducted to investigate if deletion or antagonism of the MC3R could reduce the compulsive-like eating in a preclinical model of binge eating, where a binge eating episode is elicited by the combination of food restriction and stress, trying to further characterize the role of this receptor in both homeostatic and non-homeostatic eating [86,87].

**Figure 1:**
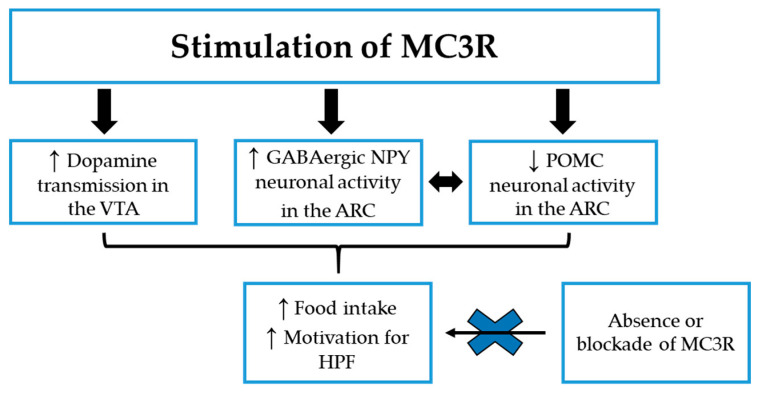
The potential MC3R mechanisms leading to increase food intake and motivation for highly palatable food (HPF) in preclinical studies. ↓: decrease; ↑: increase; ARC: Arcuate nucleus of the hypothalamus; HPF: Highly palatable food; MC3R: Melanocortin-3 receptor; NPY: neuropeptide Y; POMC: Pro-opiomelanocortin; VTA: Ventral Tegmental Area.

The information obtained from studies with MC3R KO mice are in accordance with the putative role of the MC3R as an inhibitory autoreceptor on POMC neurons [49,88,89], where α- and γ-MSH, released by POMC nerve terminals within the ARC, are supposed to regulate the activity of POMC neurons through activation of MC3R subtypes, and studies with selective MC3R agonists confirmed this observation. Indeed, Marks et al. found that stimulation of the MC3R, by peripheral administration of the selective MC3R agonist [D-trp8]-γ-MSH, results in the inhibition of POMC neuronal activity, which in turn leads to an increase in food intake in WT mice, while having no effect on feeding in MC3R KO littermates [90]. The suppression of POMC neuronal activity, after injection of [D-trp8]-γ-MSH, was demonstrated to be a consequence of an increased inhibitory synaptic transmission, due to the activation of GABAergic NPY neurons in the ARC, releasing GABA on POMC neurons [88,89]. Considering the highly potent orexigenic activity of NPY [91], this can explain the observed increase in food intake after stimulation of the MC3R [90], as reported in Figure 1.

However, the study by Marks et al. had the limitation of investigating the effect of the MC3R agonist only by a peripheral administration, not clearly explaining if the results obtained were due to a peripheral or central action. Subsequently, Lee et al., using a rat model, obtained a similar finding, analyzing the effect of the same compound directly injected in the CNS, through intracerebroventricular (i.c.v.) injections, resulting in an increased food intake in treated rats, confirming a central mechanism of action [92]. Interestingly, it was examined if antagonism at MC3R would have the opposite effect, inhibiting feeding, but a strange result was obtained, observing that the MC3R antagonist PG-932, at a low dose, suppressed food intake, while at a higher dose significantly increased food consumption and body weight. These effects could be explained with the possible antagonism profile of PG-932 even at MC4R, when injected at high doses in rats [92].

Taken together, these studies confirm that the MC3Rs, despite their functions are still not completely understood, could represent important targets for the treatment of obesity and could also play a role in the aberrant feeding patterns that characterize eating disorders.

#### 3.1.2. MC3R: Preclinical Studies in Food Reward

The melanocortin system interacts with several nuclei of the brain and neural circuits, among which, one of the most relevant in the control of food intake and body weight is the mesocorticolimbic dopamine system [93], connecting the VTA with the Nac, amygdala and PFC, regions particularly involved in reward, motivational processes and food consumption [94,95,96]. Dopamine has an essential role in food intake and reward, and thus, it is supposed that the melanocortin system can also influence feeding by modulating dopamine transmission in areas that are implicated in eating behaviors, satiety perception and reward processes. Indeed, α-MSH may affect food intake and reward, principally regulating dopamine neuronal activity in the VTA, which is part of the mesolimbic system that includes dopamine cells of the VTA projecting to the NAc [97], a key region for the reinforcing properties of highly palatable food (HPF). HPF, which consists of aliments rich in fat, sugar or both, is a potent reward and has been demonstrated to induce dopamine transmission in the NAc in both human and animal studies, increasing motivation to overconsume this type of food [94,95]. It is well documented that intra-VTA injections of α-MSH stimulate dopamine release in the NAc and dopamine-related behaviors, confirming that α-MSH increases dopamine neuronal activity in the VTA [98,99,100,101], and that POMC and AgRP neurons send projections to the VTA [102,103]. In this brain region, there is expression of both MC3Rs and MC4Rs in dopamine and non-dopamine neurons, but MC3Rs are expressed at a much higher level, compared to MC4Rs [22,46,74,104]. Conversely, the NAc shell shows a prominent concentration of MC4Rs that are found on both D1 and D2 receptor-expressing neurons [23], suggesting a differential action of MCRs on dopamine signaling in these brain areas. In light of the high expression of the MC3Rs in the VTA, the role of these receptors in the hedonic aspect of food intake was evaluated via activation of the reward circuitry. Accordingly, MC3R KO female mice, in a sucrose preference test, showed a significant reduction in the sucrose solution intake at all concentrations used (ranged from 1 to 2%), relative to WT littermates, and this was also accompanied by a decrease in sucrose preference at concentration of 1% [74]. Given the critical role of an intact VTA for sucrose preference and intake [105,106], and considering the high concentration of the MC3Rs in this region, it was hypothesized that the defect in sucrose intake in MC3R KO female mice was due to MC3R-related alterations in dopaminergic signaling in the VTA. Deletion of MC3Rs in mice was accompanied by changes in dopamine levels and its metabolites, DOPAC and homovanillic acid, in the VTA, but, interestingly, these parameters were restored in ovariectomized mice, suggesting an interaction between the melanocortin system and estrogens in the regulation of midbrain dopamine levels [74], a factor that could have an impact on food intake, taking into account the important relationship between ovarian hormones and emotional eating and binge eating, in both rodents and humans [107,108,109,110].

A following study, using MC3R^tm1Butl^ (MC3R^TB/TB^) mice, the strain in which the expression of MC3R is suppressed by insertion of a loxP-flanked transcription blocker (TB) into the genes 5′ UTR [8], reported that the absence of MC3R signaling reduced self-administration of food reward (20 mg chocolate flavored food pellet) under a progressive ratio protocol in mice subjected to caloric restriction. The result of this study suggests that the motivation to obtain a food reward in MC3R^TB/TB^ mice might be related to conditions of negative energy balance and nutrient scarcity, considering that this behavioral phenotype was not observed in mice with the same genotype, but with ad libitum access to food [21]. Moreover, acute refeeding after fasting did not induce neuronal activity (assessed by c-fos immunoreactivity) in the NAc of MC3R^TB/TB^ mice, the region associated with reward and motivation for food [21], indicating the critical role performed by the MC3R in the appetitive responses to weight loss. Rescuing the expression of the endogenous MC3Rs in the VTA partially re-established the reduced motivation to work for food reward that characterized MC3R^TB/TB^ mice, suggesting that MC3Rs expressed in the VTA could influence motivational responses to caloric restriction and have an important function in the defense of body weight during situations of poor nutrient availability [21].

Pandit et al. observed that pharmacological stimulation of the MC3Rs in the VTA increases the motivation to consume HPF, through a mechanism that involves dopaminergic transmission. Indeed, intra-VTA injection of the selective MC3R agonist γ-MSH increased response to sucrose in rats, evaluated under a progressive ratio schedule of reinforcement, an effect demonstrated by the increased number of active lever presses for sucrose. Conversely, when rats had free access to the sucrose pellet, the same treatment did not enhance free intake of both sucrose pellet or chow, indicating that MC3R stimulation selectively increases the incentive motivation for HPF and not its actual intake [22]. In the same study, i.c.v. administration of α-MSH, a MC3R/MC4R agonist, as expected, decreased the number of active lever presses, reducing response to sucrose, but when α-MSH was co-administrated with the MC4R antagonist HS014, motivation for sucrose was enhanced, supporting the role of MC3Rs in the motivation to obtain a food reward [22]. Interestingly, pretreatment with the dopamine receptors antagonist α-flupenthixol blocked the γ-MSH increased response to sucrose, and this confirms that MC3Rs in the VTA could affect food reward in a dopamine-dependent manner [22].

The result of this study is particularly interesting because it suggests that the melancortin system could fine tune motivation for HPF, depending on the type of MCR expression in different brain nuclei, considering that MC3R signaling in the VTA promotes the motivation-enhancing effects of food rewards (see Figure 1), while MC4R signaling in the NAc shell has the opposite effect, decreasing motivation for HPF [23].

### 3.2. MC4R

#### 3.2.1. MC4R: Preclinical Studies on Food Preference and Motivation

As previously mentioned, the role of the MC4R in energy homeostasis and obesity is well established, and many preclinical and clinical studies investigated the implication of this receptor in preventing weight gain and regulating energy balance. However, it has been observed that MC4R could affect feeding behaviors also modulating the brain reward circuitry, in particular by influencing neural transmission in areas sensitive to reinforcing properties of HPF [28,111,112,113].

Indeed, central administration of the endogenous MCRs antagonist AgRP in rats has been demonstrated to preferentially increase intake of a high-fat diet, over a low-fat diet, with a mechanism involving opioid transmission, considering that Naloxone, an opioid receptor antagonist, was found to selectively counteract the consumption of high-fat pellets [112]. Additionally, a selective reduction in fat consumption was found in MC4R +/+ mice treated with intraperitoneally injection of melanotan II (MTII), a MC3R and MC4R agonist, without affecting the intake in MC4R −/− littermates and, in the same study, administration of the selective MC4R agonist (pentacyclo(d-K)-Asp-cis Apc-(d)Phe-Arg-Trp-Lys-NH2) had the same effect, suggesting that the MC4R is the necessary mediator for the reduction in fat intake [114]. When administered into the Central Amygdala, a region connected with hypothalamic areas that affect eating behavior, MTII strongly reduced the high-fat diet intake, but only moderately the low-fat or standard diet, conversely to injections of SHU-9119 and AgRP, antagonists of the MCRs, in the same brain area, that increased rat preference for the high-fat diet [115].

These findings were confirmed by the study of Tracy et al., in which rats, under operant and Pavlovian conditioning paradigms, after receiving i.c.v. injections of 1 nmol AgRP, enhanced active response to earn a peanut oil emulsion (100% fat) reinforcer, but not to obtain a sucrose (100% carbohydrate) reinforcer and increased responses to cues predictive of fat delivery [113]. These results extended previous evidence that melanocortins, via MC4Rs, are probably selective for the intake of high-fat food. Accordingly, Davis et al. observed that treatment with AgRP was able to support conditioned place preference for a high-fat diet compared to standard chow, while blocking the acquisition of place preference for sucrose pellets [111], indicating a selective reinforcement effect of melanocortin antagonism directed toward fat-rich food.

The ability of AgRP to modulate food intake is supposed to be mediated, at least in part, by its influence on dopaminergic signaling in the mesocorticolimbic dopamine circuitry, and central administration of AgRP promotes activation of c-fos immunoreactivity within tyrosine-hydroxylase midbrain dopamine neurons, indicating that melanocortin antagonists are able to elicit neuronal activation in these brain areas [111]. Furthermore, AgRP-treated rats increased dopamine turnover in the medial PFC, one of the major target of dopaminergic projections from the VTA, and it is known that dopaminergic neurons in the medial PFC respond to the positive hedonic aspect of HPF [111,116,117,118]. Activation of dopamine activity in the medial PFC could also be related to the AgRP ability to promote activation of orexin-A neurons in the lateral hypothalamus [119], strictly involved in the integration of rewarding stimuli, and orexin neurons in this area send projections to the VTA [120], which in turn could stimulate dopamine activity in the medial PFC. Orexin-A neurons are thought to principally regulate arousal, but also feeding and reward-related behaviors [120], and antagonism at the orexin-1 receptor has been demonstrated to block the compulsive-like eating episode in female rats, in a preclinical model of binge eating [121]. In light of these observations, the melanocortin system could be able to promote consumption of high-fat foods in a mechanism involving opioid, dopaminergic and orexin transmissions, and future investigation should be conducted to better understand how these neurotransmitter systems interact in order to facilitate the development of dysregulated eating behaviors. 

Subsequent studies, testing MCRs agonists and antagonists, evaluated whether a direct injection of these compounds into the VTA was able to change feeding behavior, altering the activity of the mesolimbic dopamine system. Intra-VTA administration of MTII (a non-selective MC3R/MC4R agonist) dose-dependently suppressed the intake of standard chow in male rats, conversely to the MC3R/MC4R antagonist SHU-9119, which significantly stimulated 24-h food intake. Furthermore, a prolonged blockade of MCRs with the same MCRs antagonist, chronically injected for 5 days, increased total body weight, food intake and caloric efficiency, confirming that stimulation or blockade of MCRs might influence feeding behavior, by modulation of the mesolimbic dopamine transmission [122].

Taking into account this study, it was investigated if pharmacological stimulation of the MCRs in the VTA could also affect the intake of a rewarding sugar solution, under a two-bottle choice paradigm, a procedure in which rats had access to two identical drinking bottles, one containing normal water, and the other one filled with 1, 2 and 10% sucrose solutions. Intra-VTA administration of MTII dose-dependently decreased consumption of a 1 and 2% sucrose solutions, without affecting water intake in the 24-h prolonged access paradigm, while only the highest dose of MTII (50 pmol/side) reduced intake of the more appetizing 10% sucrose solution [123]. However, MTII treatment reduced not only sugar consumption in the two-bottle choice test, but also baseline 24-h food intake, raising the question of whether the effect of MCR stimulation in the mesolimbic pathway is specific or not to the hedonic aspect of food intake over the homeostatic level [123].

Additional studies have been performed to further investigate the role of the MC4Rs in the context of food reward, using self-administration paradigms, in order to evaluate if the melanocortin system could selectively affect food motivation. In light of the high expression of the MC4Rs in the NAc shell, α-MSH (0.2 nmol) and AgRP (0.1 nmol) were directly injected in this brain area, and they, respectively, decreased and increased food self-administration of 45 mg sucrose pellets, as indicated by the number of active lever presses and reinforcers earned in the operant conditioning chambers. This effect was demonstrated to be dopamine-dependent, considering that pretreatment with the dopamine receptors antagonist α-flupentixol, attenuated both active lever presses and reinforcers earned induced by AgRP [23]. Interestingly, α-MSH and AgRP, when administered in rats with free access to the sucrose pellets, did not influence feeding of the HPF, indicating that MC4Rs in the NAc shell are selectively involved in the motivation to obtain food reward [23]. A recent study, always using self-administration of sucrose pellets, under both a fixed and a progressive ratio schedule of reinforcement, obtained a similar result, considering that stimulation of MCRs with intra-VTA injections of MTII dose-dependently reduced sucrose self-administration on both schedules, while blockade of melanocortin signaling in the same area, with the MCRs antagonist SHU-9119, increased self-administration, but only under fixed ratio protocol [124].

These studies had the limit of using compounds that are not selective for MC3R or MC4R and are not in accordance with a recent finding by Pandit et al., who reported that the selective MC3R agonist γ-MSH increased sucrose self-administration when injected in the VTA [23]. This discrepancy can be explained by the activation of distinct pathways, depending on the selectivity of the agonist that activates MC3R or MC4R [124], confirming the different roles played by these MCRs in the mesolimbic dopamine system to regulate food reward and motivation, as shown in Table 1.

Finally, from these observations, it was demonstrated that the melanocortin system is able to affect different aspects of feeding behavior (from standard chow intake to self-administration of HPF) in light of its ability to interact with many other brain pathways implicated in the control of appetite and eating. Moreover, the identification of how this system is altered in aberrant eating patterns, including binge eating behavior, would be useful for a better understanding of these disorders and the discovery of new potential treatments.

#### 3.2.2. Clinical Studies on *MC4R* Mutations

Several human studies suggested that the dysfunction of the central melanocortin system, well established in the etiology of obesity, may be a potential mechanism underlying the development of altered eating patterns, due to its contribution to food seeking and consumption, appetite, hyperphagia and body weight control.

The majority of *MC4R* mutations [10], principally including missense and synonymous mutations, have demonstrated partial or complete no activity of MC4R through in vitro study [70], and this loss of function was associated with early-onset obesity in children, manifested particularly in homozygotes rather than heterozygotes, with a higher percentage of body fat mass, increased appetite and food seeking behavior during meals and hyperphagia [70]. Indeed, obese individuals, carriers of different *MC4R* mutations, compared with obese and normal weight participants without these variants, were diagnosed with BED through the completion of a validated questionnaire, thus resulting in the co-existence between obesity and BED [15,125]. In one of the first studies, despite a large number of obese children and adolescent carriers of *MC4R* gene mutations, only one girl met criteria for BED [126]. Conversely, Branson et al. found that obese individuals, carriers of *MC4R* gene mutations, met diagnostic criteria for BED, completing a validated eating disorder questionnaire [127] based on the fourth edition of Diagnostic and Statistical Manual of Mental Disorders (DMS-IV), defining BED as the major phenotype of MC4R genetic variants [15]. However, a significant controversy surrounded these findings [128], considering that other studies did not find an association between *MC4R* mutations and episodes of binge eating [129,130], and, in addition, no differences were detected in body mass index (BMI) or specific phenotype between adult carriers and non-carriers of the *MC4R* mutations [130]. In contrast to the study of Hebebrand et al., in which there were no strong associations between BED and *MC4R* mutations, Tao et al. identified BED in obese patients with specific mutations in this receptor (T11A, F51L, T112M and M200V), without being able to explain the possible pathogenesis of the development of this eating disorder in relation to *MC4R* mutations [131]. Additionally, variability of *MC4R* gene was also investigated in non-obese patients with binge eating behavior, showing a lower presence of *MC4R* mutation in this group in contrast to obese patients; however, the study was performed in a very small number of individuals with binge eating behavior and this limitation, together with the lack of a control group, might have affected the result [132].

The variants of *MC4R* were additionally considered for their possible association with the outcomes of bariatric surgery: in the study of Potoczna et al., obese patients, carriers of *MC4R* variants that presented an aggressive form of BED, were less responsive to weight loss after laparoscopic gastric banding treatment [125], while Vallette et al. did not find an influence of these genetic mutations in weight loss and body composition after the same surgical treatment [133]. A recent study evidenced that the presence of functional variants of *MC4R* significantly affected the efficacy of different laparoscopic operations in obese Swiss patients with BED, increasing the risk of reoperation due to a failure in postoperative weight loss [134]. 

These observations have encouraged further investigations of a possible involvement of *MC4R* mutations in different eating patterns, particularly in obese subjects, to explain and document the food attitudes leading to weight gain, hedonic overeating and behavioral addiction to obtain food rewards.

Valette et al. discussed how mutations could influence the choice and the preference for macronutrients: in obese adults, carriers of different functional mutations of *MC4R*, an increased carbohydrate intake compared to fat intake was reported. In the same study, using interviews with standardized questionnaires and binge eating scales, no statistical difference was found in eating behaviors in both carriers and non-carriers of *MC4R* mutations [135].

To investigate the impact of the complete loss of function of MC4R signaling on the brain response to anticipatory food reward, van der Klaauw et al. performed functional magnetic resonance imaging (fMRI) in a small group of obese individuals with heterozygous *MC4R* mutations and in obese and lean individuals without mutations in satiated state. After seeing images of HPF, surprisingly, no group difference was found in the amygdala or orbitofrontal cortex, but a hyporesponsivity to visual food cues was reported in the dorsal and ventral striatum in obese controls, compared to the response of MC4R-deficient obese patients and lean controls [136]. The result of this study is particularly relevant, knowing that dorsal striatum is a brain region involved in compulsive food seeking behavior and BED, even in a sated state [137,138]. Indeed, the understanding of how different brain responses and behavioral factors are involved in rewarding food cues may explain the reason for the development of HPF overconsumption.

#### 3.2.3. The Polymorphism rs17782313 Nearby *MC4R* Gene and Eating Behavior

Recently, a genomewide association study (GWAS) identified several single nucleotide polymorphisms (SNPs) of the *MC4R* gene, associated with high BMI and the risk of the development of obesity [139]. Among them, the SNP rs17782313, mapping to a locus 188kb downstream from the coding sequence of *MC4R* gene region, has become increasingly relevant in relation to obesity and aberrant eating behaviors [140]. Additionally, this SNP rs17782313 seems to affect expression and function of the MC4R and it has been proposed, in several studies, as a factor leading to altered eating behavior patterns, increased vulnerability to higher BMI and changes in human brain regions, especially in women and children [141,142,143,144]. 

In the study of Qi et al., high preference and intake of nutrients rich in fat, saturated fat and partly protein, without any appetite deregulation regarding carbohydrate, were found in women carriers of this SNP compared to the non-carrier participants [142], leading to an elevated risk of severe obesity. Moreover, the following works have tried to clarify the possible implication of this *MC4R* SNP in feeding behavior: Stutzmann et al. revealed an excessive appetite in a large cohort of European populations, especially eating a large amount of food during meals with a higher frequency of snacking in children and teenagers carriers of this SNP, and a greater hunger in adults carriers of the same polymorphism [143]. Snacking is a particular dietary pattern, principally during childhood, in which energy-dense and nutrient-poor food is consumed between meals exhibiting a recurrent “snack episode”, which can be translated into a bad feeding style and a risk factor for altered eating behavior and elevated BMI [145,146].

Furthermore, another study evidenced less postprandial satiation symptoms after a fully caloric satiating meal in obese individuals, carriers of rs17782313 polymorphism, promoting to eat more frequently and to increase the caloric intake in the subsequent meal, leading to higher BMI [147]. In addition, the presence of this genotypic variant demonstrated low satiety responsiveness scores, and high scores for enjoyment of food in Chilean obese children compared to the non-carrier participants [148], assessed through the Child Eating Behavior Questionnaire (CEBQ) [149] and 19-item Three-Factor Eating Questionnaire Parent (TFEQP-19) Chilean version of the TFEQ-R18 [150]. A following study, always conducted in a Chilean population, focused on obese children carriers of SNP rs17782313 revealing, in addition to lower satiety responsiveness and elevated enjoyment of food, even an overconsumption of snacks after a standard meal, using the Eating in the Absence of Hunger (EAH) Test and the CEBQ [17]. Moreover, in a three-generation Chilean family of obese women, the presence of a genetic variant of *MC4R*, generating an amino acid substitution Thr150Ile, characterized by a decreased activity of the MC4R, led to an elevated BMI and remarkable scores of cognitive restraint (CR), uncontrolled eating (UE) and emotional eating (EE), measured by TFEQ-R18 [19]. These three parameters indicate respectively: conscious lower consumption of HPF but higher intake of vegetables and proteins in order to control BMI; the tendency to eat unhealthy food more than usual in response to external stimuli with loss of control and hunger for extreme unstoppable appetite; and, finally, the inability to resist stress events, negative emotions and mood states, which often cause binge eating episodes [150,151,152]. These paradigms were also evaluated in Chilean adults, carriers of SNP rs17782313, presenting higher EE scores compared with non-carriers, while only women showed UE, evidencing a difference between women and men with this SNP [20]. 

Recent studies in obese, overweight and normal weight Chilean children extended the evidence about the SNP rs17782313, investigating how this genetic variant affects the ingestive behaviors related to reward properties of food [153]. Eating behavior scores were calculated from the EAH Test, CEBQ, TFEQ and Food Reinforcement Value Questionnaire (RVFQ), reporting differences between gender in eating patterns, but not in elevated BMI: in obese boys, carriers of the SNP, a significantly lower reinforcing value of food was observed compared to the non-carriers; meanwhile, obese girls, carriers of this polymorphism, showed lower satiety responsiveness, and UE with respect to obese girls without the SNP. These results are in accordance with the study of Vega et al., in which Chilean obese adults showed UE, suggesting the involvement of MC4R in dopamine pathways relating to food reward [153]. The hypothesis of a possible link between dopamine and melanocortin pathways has also been proposed by Yilmaz et al., underlying that this interaction could be responsible for the results of the study, in which, through the use of several questionnaires, significant EE, food craving, elevated BMI and depressive mood in European adult carriers of SNP rs17782313 were found [144]. Furthermore, the results of the case control comparisons with a group of female participants who had Anorexia or Bulimia Nervosa did not find any evidence that linked the genetic variant with these eating disorders [154].

The evidence concerning this specific polymorphism that might contribute to overweight and altered feeding patterns is not limited to the populations mentioned above, but it has been also investigated and found in subjects of different nationalities and ethnic origins [147,155,156,157,158,159,160,161,162], where the majority of these studies addressed the vulnerability of women and children to moderate and severe obesity and aberrant eating behaviors [141,157,161,163].

Horstmann et al. suggested that the genetic variation rs17782313 could affect reward mechanisms, showing that only women, homozygous carriers of the risk SNP, demonstrated EE and Disinhibition of Eating (loss of control over feeding, possibly due to external stimuli) measured by TFEQ-R18 and TFEQ-51. Moreover, through Magnetic Resonance Imaging (MRI), a sex-specific association was found between rs17782313 and an increased gray matter volume in the right amygdala, the anterior hippocampus, the medial orbitofrontal cortex and the left and the right PFC [141], crucial regions known to be involved in eating behavior [164,165].

All the studies discussed in this section (summarized in Table 2) highlighted that the partial or total loss of MC4R function, due to *MC4R* mutations, as well as the SNP rs17782313, are positively correlated with altered appetite and dysfunctional eating patterns, promoting obesity and elevated BMI.

## 4. Melanocortin System and Stress Responses

Dieting, stress and negative affect are considered potential factors able to trigger binge eating episodes in patients with BED or Bulimia Nervosa [37,166,167]. Indeed, dieting periods are commonly observed in the history of binge eaters, but hunger alone appears to be non-sufficient to induce a compulsive-like eating, if not accompanied by conditions of stress or negative affect [168,169]. Stress has a central role in the etiology of binge eating, considering that obese individuals with BED, compared to those without, show a higher activity of the HPA axis and cortisol/corticosterone plasma level [170,171,172,173]. Additionally, higher cortisol levels, induced by stress, are able to promote a greater consumption of sweet foods [174], and are also positively correlated with the severity of binge eating [175].

The melanocortin system, principally via MC4R, has been demonstrated to play a central role in stress response and negative emotional states, including anxiety and depression [176,177], suggesting the MC4R as a possible target to treat these psychiatric conditions. In fact, MC4Rs are expressed in the limbic system, mainly in several nuclei of the amygdala, such as the central and basolateral nuclei, lateral septal nucleus, hippocampus and in the entorhinal cortex [50]; thus, the distribution of the MC4R in the brain indicates an important involvement of this receptor in promoting negative emotional states [176,177]. Moreover, the MC4R, contrary to MC3R, has been highly detected in the PVN of the hypothalamus, where it is supposed to regulate the activity of the HPA axis, via arginine vasopressin (AVP) and corticotropin releasing factor (CRF) neurons [50,176]. Initial evidence linking the MC4R and stress-related responses comes from studies in which the administration of α-MSH and ACTH in rats was able to increase grooming behavior [99,100,101,178,179], characterized by many activities directed to the animal body surface, such as face washing, body grooming, licking, scratching and genital grooming, and proposed as a rodent behavioral response to stress and novel environments [178,180]. The effect of α-MSH on grooming is principally due to its agonistic activity on MC4Rs, as demonstrated by Adan et al., who found that grooming behavior, induced by MCR agonists, was positively correlated with a greater affinity and potency for MC4R, rather than for MC3R. On the contrary, the antagonist SHU-9119 attenuated grooming induced by both melanocortins and by exposure to a novel environment [178]. This finding is further confirmed by the fact that the MC4R agonist MTII increased grooming in WT, but not in mutant rats deficient in MC4Rs, confirming that this behavior is principally mediated by MC4Rs, and not by MC3R subtypes [181].

Stress has been demonstrated to have profound effects on MC4R expression and activity in the brain. In fact, the exposure to electric foot shock stress in rats increased the expression of POMC and MC4R mRNA in the hypothalamus and in the amygdala [182], region implicated in the modulation of emotional- and fear-related behaviors [183] and binge eating episodes [184,185]. 

Furthermore, rats exposed to chronic restraint stress had increased MC4R mRNA expression in the ARC of the hypothalamus, compared to control rats, not exposed to stress [186]. The effect of stress on MC4R and on feeding behavior and appetite may be also dependent on the intensity and duration of the stressor, as supported by the study of Chagra et al., in which chronic exposure to a stress induced a significant decrease in c-fos- and MC4R-expressing cells in the ARC, indicating a shift toward more orexigenic behaviors, differently from control and acutely stressed rats [187].

Pharmacological stimulation of the MC4R is able to promote the activity of the HPA axis, as reported by the study of Von Frijtag et al., in which i.c.v. injection of ACTH1-24 (the N-terminal bioactive fragment of ACTH) in rats, significantly increased plasma concentrations of ACTH and corticosterone, an effect inhibited by pretreatment with the non-selective antagonist SHU-9119 and by the selective MC4R antagonist [D-Arg8] ACTH4-10 [188]. The influence of the melanocortin system on HPA axis tone and activity can be explained considering that MC4Rs are highly expressed in the parvocellular division of the PVN [50,189], the region in which CRF neurons are also predominantly localized and where they receive α-MSH neuronal terminals [190,191]. 

In fact, activation of MC4Rs by i.c.v. injections of α-MSH or MTII increases gene expression of CRF in the PVN [189,192] and enhances corticosterone plasma levels in rats, suggesting a functional interaction between CRF and the melanocortin system [189]. In the same study, the pretreatment with the CRF antagonist α-helical-CRH9–41 was able to prevent MTII-induced suppression of food intake, evidencing that the melanocortin system can alter endogenous CRF levels in order to modulate appetite [189]. 

A stress procedure that has been demonstrated to promote activation of the melanocortin neurons, and, consequently, of the HPA axis, is the acute restraint stress. Rats exposed to this stress had a robust c-fos mRNA expression in the medial amygdala (MeA) [18,193], a brain region with high levels of MC4Rs [50], and particularly sensitive to psychological stressors, characterized by an emotional component, such as restraint [194,195]. Lesions of the MeA result in a blunted response of the HPA axis to psychogenic stressors [196], conversely to pharmacological stimulation of the MC4R-expressing neurons in the MeA, which promotes corticosterone release [18]. Moreover, both stress-induced anorexia and corticosterone release, in response to the acute restraint stress, can be prevented by administration of a MC4R antagonist directly in the MeA [18,193]. The interaction between MC4Rs in the MeA and the CRF system is probably mediated by the efferents from the MeA to the Bed Nucleus of the Stria Terminalis (BNST), brain region enriched in CRF neurons [197], and involved in stress-induced emotional responses and activation of the HPA axis [198,199]. The BNST has been demonstrated to play a pivotal role in stress-induced binge eating for HPF, evoked by a combination of frustration stress and food restriction [200,201], and injection of a non-selective CRF receptor antagonist directly into the BNST was able to counteract this compulsive-like eating episode for HPF selectively in rats exposed to both stress and restriction [200]. These findings support the hypothesis that MC4R can also influence the activation of the HPA axis via extrahypothalamic sites, and thus could represent an important factor for the development of aberrant feeding behaviors in response to stress exposure. Consistently with this evidence, acute stress-induced release of ACTH and corticosterone, as well as neuronal activation in the PVN and MeA, were significantly attenuated in male rats with a *MC4R* mutation, producing a less functional receptor, compared to the WT littermates [202,203]. Intriguingly, it was observed that female rats with the same mutation revealed an unexpected and exaggerated acute stress-induced corticosterone release, contrary to mutant males, highlighting a difference in stress reactivity between male and female rats with the MC4R loss of function [202]. The result of this study suggests a sex-dependent responsivity in the basal HPA axis tone and acute stress-induced corticosterone in rodents with MC4R mutation [202]. Considering the heightened stress reactivity found in female rats with deficient MC4R activity [202], and that stress has been associated with EE [37,38] and binge eating behavior [38,204,205,206], it would be interesting in the future to evaluate the potential involvement of MC4R signaling in a female rat model of binge eating, in which the binge eating episode is elicited by a combination of food restriction plus stress [86,87] and by using HPF, in order to promote the aberrant feeding behavior and to increase the motivation to overconsume food [38,87,207,208,209].

## 5. Conclusions

**Figure 2:**
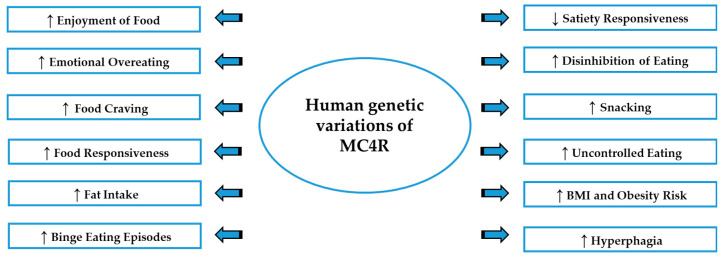
An overview of the altered eating patterns associated with the genetic variation of MC4R. ↓: decrease; ↑: increase; BMI: Body mass index; MC4R: Melanocortin-4 receptor.

The pivotal role played by melanocortin system in controlling feeding behavior, appetite, energy balance and motivation for rewarding properties of food can explain why dysfunction of this system, in both human and rodent studies, results in a breakdown of normal regulatory processes and in more vulnerability to the loss of control in food intake, possibly leading to altered eating patterns, as summarized in Figure 2. Further research needs to highlight the mechanisms driving the hyperphagia in melanocortin-associated obesity, evidencing whether the exaggerated food consumption is accompanied by the loss of behavioral control, food seeking and/or binge eating episodes. Recent studies concerning MC3R and MC4R revealed that melanocortin signaling can exert functional effects in reward-related behaviors, due the hedonic properties of HPF, which is a potent natural reinforcer, and it has been postulated that in humans, low melanocortin activity could predispose individuals to pathological overeating, developing obesity and altered feeding behavior. Finally, the consistent relationship between the MC4R and stress response can be considered an additional factor linking melanocortin signaling to binge eating episodes, given the key role of stress in the etiology of this compulsive behavior. More preclinical studies are needed to investigate the biological mechanisms underlying dysfunctional eating patterns and clarify the possible connection between MCRs and binge eating behavior.

## Figures and Tables

**Table 1:** Summary of studies regarding MC3R and MC4R on food reward and motivation.

Species	Experiment	Result	Ref.
MC3R KO vs. WT mice	Sucrose preference test	↓ sucrose intake and preference in female MC3R KO mice	[74]
MC3R^tm1Butl^ (MC3R^TB/TB^) vs. WT mice	Food self-administration under fixed and progressive ratio protocols	↓ self-administration of a food reward in MC3R^TB/TB^ mice exposed to caloric restriction	[21]
Rats	Food self-administration under fixed and progressive ratio protocols	↑ operant response, but not free access to sucrose after injection of the MC3R agonist γ-MSH	[22]
Rats	Consumption of a high-fat vs. low-fat diet	↑ intake of a high-fat diet vs. a low-fat diet after i.c.v. injection of AgRP	[112]
MC4R +/+ vs. MC4R −/− mice	Consumption of a three-choice diet (fat, protein, carbohydrate)	↓ fat intake in MC4R +/+, but not in MC4R −/− mice after injections of the MC3R/MC4R agonist MTII and the MC4R agonist (pentacyclo(D-K)-Asp-cis Apc-(D)Phe-Arg-Trp-Lys-NH2)	[114]
Rats	High-fat vs. low-fat diet paradigm	↓ the high-fat diet intake after injection of MTII in the CeA; ↑ high-fat diet consumption after the injection MCRs antagonists SHU-9119 and AgRP	[115]
Rats	Fat and sugar consumption under an operant conditioning paradigm	↑ active response to earn a peanut oil emulsion (100% fat) reinforcer, but not a sucrose (100% carbohydrate) reinforcer after i.c.v. injection of AgRP	[113]
Rats	Conditioned place preference for high-fat diet and sucrose pellets	AgRP supports conditioned place preference for a high-fat diet, while blocks the acquisition of place preference for sucrose pellets	[111]
Rats	Consumption of standard chow	↓ intake of standard chow after intra-VTA injection of MTII; ↑ 24-h food intake with SHU-9119	[122]
Rats	Two-bottle choice paradigm for a sucrose solution	↓ consumption of a 1 and 2% sucrose solutions with intra-VTA injections of MTII; ↓ intake of the more appetizing 10% sucrose solution only at the highest dose of MTII	[39]
Rats	Food self-administration under fixed and progressive ratio protocols	↓ operant response with α-MSH and ↑ operant response with AgRP injected in the NAc shell; no influence on free consumption of sucrose pellets	[23]
Rats	Food self-administration under fixed and progressive ratio protocols	↓ sucrose self-administration on both fixed and progressive ratio schedules with intra-VTA injections of MTII; ↑ self-administration, only under fixed ratio protocols with SHU-9119	[124]

↓: decrease; ↑: increase; AgRP: Agouti-related protein; α-MSH: α-melanocyte-stimulating hormone; γ-MSH: γ-melanocyte-stimulating hormone; CeA: Central Amygdala; i.c.v.: Intracerebroventricular; KO: Knock-out; MCRs: Melanocortin receptors; MC3R: Melanocortin-3 receptor; MC4R: Melanocortin-4 receptor; MTII: Melanotan II; NAc: Nucleus Accumbens; VTA: Ventral Tegmental Area; WT: Wild-Type.

**Table 2:** *MC4R* variant rs17782313 and manifestation of altered eating behavioral phenotype.

Subjects with the *MC4R* Variant rs17782313	Result	Ref.
Normal weight vs. obese children of both sexes	Obese children present high scores of Enjoyment of Food, Emotional Overeating, Food Responsiveness and lower Satiety Responsiveness	[17]
Adult participants	In both genders high scores of Emotional Eating associated with BMI were found, while only in women the Uncontrolled Eating scores were associated with BMI.	[20]
Healthy adult volunteers	Only women, especially homozygous carriers of *MC4R* variant rs17782313, demonstrated Emotional Eating and Disinhibition of Eating.	[141]
Adult women	Women had significantly higher intake of energy from fat, compared to carbohydrate.	[142]
Children, teenagers and adults	Children and teenagers presented snacking and eating large amounts of food during meals. Adults presented a greater hunger score	[143]
Adults between the ages of 24 and 50 years	Overeating behaviors, Emotional Eating and Food Cravings.	[144]
Overweight or obese participants	Less postprandial satiation symptoms after a fully caloric meal	[147]
Obese children	Low Satiety Responsiveness scores and high scores for the Enjoyment of Food	[148]
Obese, overweight and normal weight children	In obese girls were found significant lower scores of the Satiety Responsiveness and higher scores of the Uncontrolled Eating	[153]
Women	Association with increased BMI and obesity	[160,161,163]
Lean, overweight, and obese children	Association with increased BMI and obesity	[155,159]
Normal weight vs. obese adults	Association with increased BMI and obesity	[156,162]
Normal weight vs. obese adults	Association with increased BMI and obesity and a significant higher intake of energy from fat compared to carbohydrate.	[158]

BMI: Body Mass Index; MC4R: Melanocortin-4 receptor.

## References

[1] Cordain L., Eaton S.B., Sebastian A., Mann N., Lindeberg S., Watkins B.A., O’Keefe J.H., Brand-Miller J. (2005). Origins and evolution of the Western diet: Health implications for the 21st century. Am. J. Clin. Nutr..

[2] Giudetti A.M., Micioni Di Bonaventura M.V., Ferramosca A., Longo S., Micioni Di Bonaventura E., Friuli M., Romano A., Gaetani S., Cifani C. (2020). Brief daily access to cafeteria-style diet impairs hepatic metabolism even in the absence of excessive body weight gain in rats. FASEB J..

[3] Cone R.D. (1999). The Central Melanocortin System and Energy Homeostasis. Trends Endocrinol. Metab..

[4] Cone R.D. (2006). Studies on the physiological functions of the melanocortin system. Endocr. Rev..

[5] Girardet C., Butler A.A. (2014). Neural melanocortin receptors in obesity and related metabolic disorders. Biochim. Biophys. Acta.

[6] Butler A.A. (2006). The melanocortin system and energy balance. Peptides.

[7] Fan W., Boston B.A., Kesterson R.A., Hruby V.J., Cone R.D. (1997). Role of melanocortinergic neurons in feeding and the agouti obesity syndrome. Nature.

[8] Beckers S., Zegers D., de Freitas F., Mertens I.L., Van Gaal L.F., Van Hul W. (2011). Association study of MC4R with complex obesity and replication of the rs17782313 association signal. Mol. Genet. Metab..

[9] Benoit S., Schwartz M., Baskin D., Woods S.C., Seeley R.J. (2000). CNS melanocortin system involvement in the regulation of food intake. Horm. Behav..

[10] Hinney A., Volckmar A.L., Knoll N. (2013). Melanocortin-4 receptor in energy homeostasis and obesity pathogenesis. Prog. Mol. Biol. Transl. Sci..

[11] Huszar D., Lynch C.A., Fairchild-Huntress V., Dunmore J.H., Fang Q., Berkemeier L.R., Gu W., Kesterson R.A., Boston B.A., Cone R.D. (1997). Targeted disruption of the melanocortin-4 receptor results in obesity in mice. Cell.

[12] Kleinendorst L., van Haelst M.M., van den Akker E.L.T. (2019). Genetics of Obesity. Exp. Suppl..

[13] Vaisse C., Clement K., Guy-Grand B., Froguel P. (1998). A frameshift mutation in human MC4R is associated with a dominant form of obesity. Nat. Genet..

[14] Yeo G.S., Farooqi I.S., Aminian S., Halsall D.J., Stanhope R.G., O’Rahilly S. (1998). A frameshift mutation in MC4R associated with dominantly inherited human obesity. Nat. Genet..

[15] Branson R., Potoczna N., Kral J.G., Lentes K.U., Hoehe M.R., Horber F.F. (2003). Binge eating as a major phenotype of melanocortin 4 receptor gene mutations. N. Engl. J. Med..

[16] Butler A.A., Marks D.L., Fan W., Kuhn C.M., Bartolome M., Cone R.D. (2001). Melanocortin-4 receptor is required for acute homeostatic responses to increased dietary fat. Nat. Neurosci..

[17] Ho-Urriola J., Guzman-Guzman I.P., Smalley S.V., Gonzalez A., Weisstaub G., Dominguez-Vasquez P., Valladares M., Amador P., Hodgson M.I., Obregon A.M. (2014). Melanocortin-4 receptor polymorphism rs17782313: Association with obesity and eating in the absence of hunger in Chilean children. Nutrition.

[18] Liu J., Garza J.C., Li W., Lu X.Y. (2013). Melanocortin-4 receptor in the medial amygdala regulates emotional stress-induced anxiety-like behaviour, anorexia and corticosterone secretion. Int. J. Neuropsychopharmacol..

[19] Santos J.L., Amador P., Valladares M., Albala C., Martinez J.A., Marti A. (2008). Obesity and eating behaviour in a three-generation Chilean family with carriers of the Thrl50Ile mutation in the melanocortin-4 receptor gene. J. Physiol. Biochem..

[20] Vega J.A., Salazar G., Hodgson M.I., Cataldo L.R., Valladares M., Obregon A.M., Santos J.L. (2016). Melanocortin-4 Receptor Gene Variation Is Associated with Eating Behavior in Chilean Adults. Ann. Nutr. Metab..

[21] Mavrikaki M., Girardet C., Kern A., Faruzzi Brantley A., Miller C.A., Macarthur H., Marks D.L., Butler A.A. (2016). Melanocortin-3 receptors in the limbic system mediate feeding-related motivational responses during weight loss. Mol. Metab..

[22] Pandit R., Omrani A., Luijendijk M.C., de Vrind V.A., Van Rozen A.J., Ophuis R.J., Garner K., Kallo I., Ghanem A., Liposits Z. (2016). Melanocortin 3 Receptor Signaling in Midbrain Dopamine Neurons Increases the Motivation for Food Reward. Neuropsychopharmacology.

[23] Pandit R., van der Zwaal E.M., Luijendijk M.C., Brans M.A., van Rozen A.J., Oude Ophuis R.J., Vanderschuren L.J., Adan R.A., la Fleur S.E. (2015). Central melanocortins regulate the motivation for sucrose reward. PLoS ONE.

[24] De Franca G.V., Gigante D.P., Olinto M.T. (2014). Binge eating in adults: Prevalence and association with obesity, poor self-rated health status and body dissatisfaction. Public Health Nutr..

[25] Hudson J.I., Hiripi E., Pope H.G., Kessler R.C. (2007). The prevalence and correlates of eating disorders in the National Comorbidity Survey Replication. Biol. Psychiatry.

[26] Kessler R.C., Berglund P.A., Chiu W.T., Deitz A.C., Hudson J.I., Shahly V., Aguilar-Gaxiola S., Alonso J., Angermeyer M.C., Benjet C. (2013). The prevalence and correlates of binge eating disorder in the World Health Organization World Mental Health Surveys. Biol. Psychiatry.

[27] Kishi T., Aschkenasi C.J., Lee C.E., Mountjoy K.G., Saper C.B., Elmquist J.K. (2003). Expression of melanocortin 4 receptor mRNA in the central nervous system of the rat. J. Comp. Neurol..

[28] Yoon Y.R., Baik J.H. (2015). Melanocortin 4 Receptor and Dopamine D2 Receptor Expression in Brain Areas Involved in Food Intake. Endocrinol. Metab..

[29] American Psychiatric Association (2013). Diagnostic and Statistical Manual of Mental Disorders: DSM-5.

[30] Guerdjikova A.I., Mori N., Casuto L.S., McElroy S.L. (2019). Update on Binge Eating Disorder. Med. Clin. N. Am..

[31] Ulfvebrand S., Birgegard A., Norring C., Hogdahl L., von Hausswolff-Juhlin Y. (2015). Psychiatric comorbidity in women and men with eating disorders results from a large clinical database. Psychiatry Res..

[32] Yanovski S.Z. (1993). Binge eating disorder: Current knowledge and future directions. Obes. Res..

[33] Schag K., Schonleber J., Teufel M., Zipfel S., Giel K.E. (2013). Food-related impulsivity in obesity and binge eating disorder—A systematic review. Obes. Rev..

[34] Manwaring J.L., Green L., Myerson J., Strube M.J., Wilfley D.E. (2011). Discounting of Various types of rewards by women with and without binge eating Disorder: Evidence for general rather than specific Differences. Psychol. Rec..

[35] Moore C.F., Sabino V., Koob G.F., Cottone P. (2017). Neuroscience of Compulsive Eating Behavior. Front. Neurosci..

[36] Zeeck A., Stelzer N., Linster H.W., Joos A., Hartmann A. (2011). Emotion and eating in binge eating disorder and obesity. Eur. Eat. Disord. Rev..

[37] Adam T.C., Epel E.S. (2007). Stress, eating and the reward system. Physiol. Behav..

[38] Micioni Di Bonaventura M.V., Micioni Di Bonaventura E., Polidori C., Cifani C., Frank G.K.W., Berner L.A. (2020). Preclinical Models of Stress and Environmental Influences on Binge Eating. Binge Eating: A Transdiagnostic Psychopathology.

[39] Rosenbaum D.L., White K.S. (2015). The relation of anxiety, depression, and stress to binge eating behavior. J. Health Psychol..

[40] He J., Cai Z., Fan X. (2017). Prevalence of binge and loss of control eating among children and adolescents with overweight and obesity: An exploratory meta-analysis. Int. J. Eat. Disord..

[41] Bagnol D., Lu X.Y., Kaelin C.B., Day H.E., Ollmann M., Gantz I., Akil H., Barsh G.S., Watson S.J. (1999). Anatomy of an endogenous antagonist: Relationship between Agouti-related protein and proopiomelanocortin in brain. J. Neurosci..

[42] Ollmann M.M., Wilson B.D., Yang Y.K., Kerns J.A., Chen Y., Gantz I., Barsh G.S. (1997). Antagonism of central melanocortin receptors in vitro and in vivo by agouti-related protein. Science.

[43] Gantz I., Konda Y., Tashiro T., Shimoto Y., Miwa H., Munzert G., Watson S.J., DelValle J., Yamada T. (1993). Molecular cloning of a novel melanocortin receptor. J. Biol. Chem..

[44] Gantz I., Miwa H., Konda Y., Shimoto Y., Tashiro T., Watson S.J., DelValle J., Yamada T. (1993). Molecular cloning, expression, and gene localization of a fourth melanocortin receptor. J. Biol. Chem..

[45] Mountjoy K.G., Robbins L.S., Mortrud M.T., Cone R.D. (1992). The cloning of a family of genes that encode the melanocortin receptors. Science.

[46] Roselli-Rehfuss L., Mountjoy K.G., Robbins L.S., Mortrud M.T., Low M.J., Tatro J.B., Entwistle M.L., Simerly R.B., Cone R.D. (1993). Identification of a receptor for gamma melanotropin and other proopiomelanocortin peptides in the hypothalamus and limbic system. Proc. Natl. Acad. Sci. USA.

[47] Adan R.A., Cone R.D., Burbach J.P., Gispen W.H. (1994). Differential effects of melanocortin peptides on neural melanocortin receptors. Mol. Pharmacol..

[48] Mountjoy K.G. (2010). Distribution and function of melanocortin receptors within the brain. Adv. Exp. Med. Biol..

[49] Jegou S., Boutelet I., Vaudry H. (2000). Melanocortin-3 receptor mRNA expression in pro-opiomelanocortin neurones of the rat arcuate nucleus. J. Neuroendocrinol..

[50] Mountjoy K.G., Mortrud M.T., Low M.J., Simerly R.B., Cone R.D. (1994). Localization of the melanocortin-4 receptor (MC4-R) in neuroendocrine and autonomic control circuits in the brain. Mol. Endocrinol..

[51] Mountjoy K.G., Wild J.M. (1998). Melanocortin-4 receptor mRNA expression in the developing autonomic and central nervous systems. Dev. Brain Res..

[52] Small C.J., Kim M.S., Stanley S.A., Mitchell J.R., Murphy K., Morgan D.G., Ghatei M.A., Bloom S.R. (2001). Effects of chronic central nervous system administration of agouti-related protein in pair-fed animals. Diabetes.

[53] Beckers S., Zegers D., Van Gaal L.F., Van Hul W. (2009). The role of the leptin-melanocortin signalling pathway in the control of food intake. Crit. Rev. Eukaryot. Gene Expr..

[54] Fan W., Dinulescu D.M., Butler A.A., Zhou J., Marks D.L., Cone R.D. (2000). The central melanocortin system can directly regulate serum insulin levels. Endocrinology.

[55] Williams K.W., Scott M.M., Elmquist J.K. (2011). Modulation of the central melanocortin system by leptin, insulin, and serotonin: Co-ordinated actions in a dispersed neuronal network. Eur. J. Pharmacol..

[56] Micioni Di Bonaventura M.V., Micioni Di Bonaventura E., Cifani C., Polidori C. (2019). N/OFQ-NOP System in Food Intake. Handb. Exp. Pharmacol..

[57] Cifani C., Guerrini R., Massi M., Polidori C. (2006). Chronic intracerebroventricular infusion of nociceptin/orphanin FQ increases food and ethanol intake in alcohol-preferring rats. Peptides.

[58] Bomberg E.M., Grace M.K., Levine A.S., Olszewski P.K. (2006). Functional interaction between nociceptin/orphanin FQ and alpha-melanocyte-stimulating hormone in the regulation of feeding. Peptides.

[59] Filaferro M., Ruggieri V., Novi C., Calo G., Cifani C., Micioni Di Bonaventura M.V., Sandrini M., Vitale G. (2014). Functional antagonism between nociceptin/orphanin FQ and corticotropin-releasing factor in rat anxiety-related behaviors: Involvement of the serotonergic system. Neuropeptides.

[60] Vitale G., Filaferro M., Micioni Di Bonaventura M.V., Ruggieri V., Cifani C., Guerrini R., Simonato M., Zucchini S. (2017). Effects of [Nphe(1), Arg(14), Lys(15)] N/OFQ-NH2 (UFP-101), a potent NOP receptor antagonist, on molecular, cellular and behavioural alterations associated with chronic mild stress. J. Psychopharmacol..

[61] Hardaway J.A., Jensen J., Kim M., Mazzone C.M., Sugam J.A., Diberto J.F., Lowery-Gionta E.G., Hwa L.S., Pleil K.E., Bulik C.M. (2016). Nociceptin receptor antagonist SB 612111 decreases high fat diet binge eating. Behav. Brain Res..

[62] Micioni Di Bonaventura M.V., Ubaldi M., Liberati S., Ciccocioppo R., Massi M., Cifani C. (2013). Caloric restriction increases the sensitivity to the hyperphagic effect of nociceptin/orphanin FQ limiting its ability to reduce binge eating in female rats. Psychopharmacology.

[63] Pucci M., Micioni Di Bonaventura M.V., Giusepponi M.E., Romano A., Filaferro M., Maccarrone M., Ciccocioppo R., Cifani C., D’Addario C. (2016). Epigenetic regulation of nociceptin/orphanin FQ and corticotropin-releasing factor system genes in frustration stress-induced binge-like palatable food consumption. Addict. Biol..

[64] Statnick M.A., Chen Y., Ansonoff M., Witkin J.M., Rorick-Kehn L., Suter T.M., Song M., Hu C., Lafuente C., Jimenez A. (2016). A Novel Nociceptin Receptor Antagonist LY2940094 Inhibits Excessive Feeding Behavior in Rodents: A Possible Mechanism for the Treatment of Binge Eating Disorder. J. Pharmacol. Exp. Ther..

[65] Butler A.A., Kesterson R.A., Khong K., Cullen M.J., Pelleymounter M.A., Dekoning J., Baetscher M., Cone R.D. (2000). A unique metabolic syndrome causes obesity in the melanocortin-3 receptor-deficient mouse. Endocrinology.

[66] Chen A.S., Marsh D.J., Trumbauer M.E., Frazier E.G., Guan X.M., Yu H., Rosenblum C.I., Vongs A., Feng Y., Cao L. (2000). Inactivation of the mouse melanocortin-3 receptor results in increased fat mass and reduced lean body mass. Nat. Genet..

[67] Ste Marie L., Miura G.I., Marsh D.J., Yagaloff K., Palmiter R.D. (2000). A metabolic defect promotes obesity in mice lacking melanocortin-4 receptors. Proc. Natl. Acad. Sci. USA.

[68] Sutton G.M., Trevaskis J.L., Hulver M.W., McMillan R.P., Markward N.J., Babin M.J., Meyer E.A., Butler A.A. (2006). Diet-genotype interactions in the development of the obese, insulin-resistant phenotype of C57BL/6J mice lacking melanocortin-3 or -4 receptors. Endocrinology.

[69] Begriche K., Girardet C., McDonald P., Butler A.A. (2013). Melanocortin-3 receptors and metabolic homeostasis. Prog. Mol. Biol. Transl. Sci..

[70] Farooqi I.S., Keogh J.M., Yeo G.S., Lank E.J., Cheetham T., O’Rahilly S. (2003). Clinical spectrum of obesity and mutations in the melanocortin 4 receptor gene. N. Engl. J. Med..

[71] Hinney A., Schmidt A., Nottebom K., Heibult O., Becker I., Ziegler A., Gerber G., Sina M., Gorg T., Mayer H. (1999). Several mutations in the melanocortin-4 receptor gene including a nonsense and a frameshift mutation associated with dominantly inherited obesity in humans. J. Clin. Endocrinol. Metab..

[72] Schalin-Jantti C., Valli-Jaakola K., Oksanen L., Martelin E., Laitinen K., Krusius T., Mustajoki P., Heikinheimo M., Kontula K. (2003). Melanocortin-3-receptor gene variants in morbid obesity. Int. J. Obes. Relat. Metab. Disord..

[73] Begriche K., Sutton G.M., Butler A.A. (2011). Homeostastic and non-homeostatic functions of melanocortin-3 receptors in the control of energy balance and metabolism. Physiol. Behav..

[74] Lippert R.N., Ellacott K.L., Cone R.D. (2014). Gender-specific roles for the melanocortin-3 receptor in the regulation of the mesolimbic dopamine system in mice. Endocrinology.

[75] Duncan A., Heyer M.P., Ishikawa M., Caligiuri S.P.B., Liu X.A., Chen Z., Micioni Di Bonaventura M.V., Elayouby K.S., Ables J.L., Howe W.M. (2019). Habenular TCF7L2 links nicotine addiction to diabetes. Nature.

[76] Zhang Y., Kilroy G.E., Henagan T.M., Prpic-Uhing V., Richards W.G., Bannon A.W., Mynatt R.L., Gettys T.W. (2005). Targeted deletion of melanocortin receptor subtypes 3 and 4, but not CART, alters nutrient partitioning and compromises behavioral and metabolic responses to leptin. FASEB J..

[77] Lee B., Koo J., Yun Jun J., Gavrilova O., Lee Y., Seo A.Y., Taylor-Douglas D.C., Adler-Wailes D.C., Chen F., Gardner R. (2016). A mouse model for a partially inactive obesity-associated human MC3R variant. Nat. Commun..

[78] Feng N., Young S.F., Aguilera G., Puricelli E., Adler-Wailes D.C., Sebring N.G., Yanovski J.A. (2005). Co-occurrence of two partially inactivating polymorphisms of MC3R is associated with pediatric-onset obesity. Diabetes.

[79] Lee Y.S., Poh L.K., Kek B.L., Loke K.Y. (2007). The role of melanocortin 3 receptor gene in childhood obesity. Diabetes.

[80] Savastano D.M., Tanofsky-Kraff M., Han J.C., Ning C., Sorg R.A., Roza C.A., Wolkoff L.E., Anandalingam K., Jefferson-George K.S., Figueroa R.E. (2009). Energy intake and energy expenditure among children with polymorphisms of the melanocortin-3 receptor. Am. J. Clin. Nutr..

[81] Butler A.A., Girardet C., Mavrikaki M., Trevaskis J.L., Macarthur H., Marks D.L., Farr S.A. (2017). A Life without Hunger: The Ups (and Downs) to Modulating Melanocortin-3 Receptor Signaling. Front. Neurosci..

[82] Sutton G.M., Perez-Tilve D., Nogueiras R., Fang J., Kim J.K., Cone R.D., Gimble J.M., Tschop M.H., Butler A.A. (2008). The melanocortin-3 receptor is required for entrainment to meal intake. J. Neurosci..

[83] Begriche K., Marston O.J., Rossi J., Burke L.K., McDonald P., Heisler L.K., Butler A.A. (2012). Melanocortin-3 receptors are involved in adaptation to restricted feeding. Genes Brain Behav..

[84] Girardet C., Mavrikaki M.M., Stevens J.R., Miller C.A., Marks D.L., Butler A.A. (2017). Melanocortin-3 receptors expressed in Nkx2.1(+ve) neurons are sufficient for controlling appetitive responses to hypocaloric conditioning. Sci. Rep..

[85] Renquist B.J., Murphy J.G., Larson E.A., Olsen D., Klein R.F., Ellacott K.L., Cone R.D. (2012). Melanocortin-3 receptor regulates the normal fasting response. Proc. Natl. Acad. Sci. USA.

[86] Cifani C., Polidori C., Melotto S., Ciccocioppo R., Massi M. (2009). A preclinical model of binge eating elicited by yo-yo dieting and stressful exposure to food: Effect of sibutramine, fluoxetine, topiramate, and midazolam. Psychopharmacology.

[87] Hagan M.M., Wauford P.K., Chandler P.C., Jarrett L.A., Rybak R.J., Blackburn K. (2002). A new animal model of binge eating: Key synergistic role of past caloric restriction and stress. Physiol. Behav..

[88] Cowley M.A., Cone R., Enriori P., Louiselle I., Williams S.M., Evans A.E. (2003). Electrophysiological actions of peripheral hormones on melanocortin neurons. Ann. N. Y. Acad. Sci..

[89] Cowley M.A., Smart J.L., Rubinstein M., Cerdan M.G., Diano S., Horvath T.L., Cone R.D., Low M.J. (2001). Leptin activates anorexigenic POMC neurons through a neural network in the arcuate nucleus. Nature.

[90] Marks D.L., Hruby V., Brookhart G., Cone R.D. (2006). The regulation of food intake by selective stimulation of the type 3 melanocortin receptor (MC3R). Peptides.

[91] Gehlert D.R. (1999). Role of hypothalamic neuropeptide Y in feeding and obesity. Neuropeptides.

[92] Lee M., Kim A., Conwell I.M., Hruby V., Mayorov A., Cai M., Wardlaw S.L. (2008). Effects of selective modulation of the central melanocortin-3-receptor on food intake and hypothalamic POMC expression. Peptides.

[93] West K.S., Lu C., Olson D.P., Roseberry A.G. (2019). Alpha-melanocyte stimulating hormone increases the activity of melanocortin-3 receptor-expressing neurons in the ventral tegmental area. J. Physiol..

[94] Kenny P.J. (2011). Reward mechanisms in obesity: New insights and future directions. Neuron.

[95] Volkow N.D., Wang G.J., Baler R.D. (2011). Reward, dopamine and the control of food intake: Implications for obesity. Trends Cogn. Sci..

[96] Nair S.G., Navarre B.M., Cifani C., Pickens C.L., Bossert J.M., Shaham Y. (2011). Role of dorsal medial prefrontal cortex dopamine D1-family receptors in relapse to high-fat food seeking induced by the anxiogenic drug yohimbine. Neuropsychopharmacology.

[97] Wise R.A. (2009). Roles for nigrostriatal—Not just mesocorticolimbic—Dopamine in reward and addiction. Trends Neurosci..

[98] Lindblom J., Opmane B., Mutulis F., Mutule I., Petrovska R., Klusa V., Bergstrom L., Wikberg J.E. (2001). The MC4 receptor mediates alpha-MSH induced release of nucleus accumbens dopamine. Neuroreport.

[99] Sanchez M.S., Barontini M., Armando I., Celis M.E. (2001). Correlation of increased grooming behavior and motor activity with alterations in nigrostriatal and mesolimbic catecholamines after alpha-melanotropin and neuropeptide glutamine-isoleucine injection in the rat ventral tegmental area. Cell. Mol. Neurobiol..

[100] Torre E., Celis M.E. (1986). Alpha-MSH injected into the substantia nigra or intraventricularly alters behavior and the striatal dopaminergic activity. Neurochem. Int..

[101] Torre E., Celis M.E. (1988). Cholinergic mediation in the ventral tegmental area of alpha-melanotropin induced excessive grooming: Changes of the dopamine activity in the nucleus accumbens and caudate putamen. Life Sci..

[102] Dietrich M.O., Bober J., Ferreira J.G., Tellez L.A., Mineur Y.S., Souza D.O., Gao X.B., Picciotto M.R., Araujo I., Liu Z.W. (2012). AgRP neurons regulate development of dopamine neuronal plasticity and nonfood-associated behaviors. Nat. Neurosci..

[103] King C.M., Hentges S.T. (2011). Relative number and distribution of murine hypothalamic proopiomelanocortin neurons innervating distinct target sites. PLoS ONE.

[104] Liu H., Kishi T., Roseberry A.G., Cai X., Lee C.E., Montez J.M., Friedman J.M., Elmquist J.K. (2003). Transgenic mice expressing green fluorescent protein under the control of the melanocortin-4 receptor promoter. J. Neurosci..

[105] Martinez-Hernandez J., Lanuza E., Martinez-Garcia F. (2006). Selective dopaminergic lesions of the ventral tegmental area impair preference for sucrose but not for male sexual pheromones in female mice. Eur. J. Neurosci..

[106] Shibata R., Kameishi M., Kondoh T., Torii K. (2009). Bilateral dopaminergic lesions in the ventral tegmental area of rats influence sucrose intake, but not umami and amino acid intake. Physiol. Behav..

[107] Culbert K.M., Racine S.E., Klump K.L. (2016). Hormonal Factors and Disturbances in Eating Disorders. Curr. Psychiatry Rep..

[108] Edler C., Lipson S.F., Keel P.K. (2007). Ovarian hormones and binge eating in bulimia nervosa. Psychol. Med..

[109] Micioni Di Bonaventura M.V., Lutz T.A., Romano A., Pucci M., Geary N., Asarian L., Cifani C. (2017). Estrogenic suppression of binge-like eating elicited by cyclic food restriction and frustrative-nonreward stress in female rats. Int. J. Eat. Disord..

[110] Alboni S., Micioni Di Bonaventura M.V., Benatti C., Giusepponi M.E., Brunello N., Cifani C. (2017). Hypothalamic expression of inflammatory mediators in an animal model of binge eating. Behav. Brain Res..

[111] Davis J.F., Choi D.L., Shurdak J.D., Krause E.G., Fitzgerald M.F., Lipton J.W., Sakai R.R., Benoit S.C. (2011). Central melanocortins modulate mesocorticolimbic activity and food seeking behavior in the rat. Physiol. Behav..

[112] Hagan M.M., Rushing P.A., Benoit S.C., Woods S.C., Seeley R.J. (2001). Opioid receptor involvement in the effect of AgRP- (83-132) on food intake and food selection. Am. J. Physiol. Regul. Integr. Comp. Physiol..

[113] Tracy A.L., Clegg D.J., Johnson J.D., Davidson T.L., Benoit S.C. (2008). The melanocortin antagonist AgRP (83-132) increases appetitive responding for a fat, but not a carbohydrate, reinforcer. Pharmacol. Biochem. Behav..

[114] Samama P., Rumennik L., Grippo J.F. (2003). The melanocortin receptor MCR4 controls fat consumption. Regul. Pept..

[115] Boghossian S., Park M., York D.A. (2010). Melanocortin activity in the amygdala controls appetite for dietary fat. Am. J. Physiol. Regul. Integr. Comp. Physiol..

[116] Bassareo V., Di Chiara G. (1997). Differential influence of associative and nonassociative learning mechanisms on the responsiveness of prefrontal and accumbal dopamine transmission to food stimuli in rats fed ad libitum. J. Neurosci..

[117] Botticelli L., Micioni Di Bonaventura E., Del Bello F., Giorgioni G., Piergentili A., Romano A., Quaglia W., Cifani C., Micioni Di Bonaventura M.V. (2020). Underlying Susceptibility to Eating Disorders and Drug Abuse: Genetic and Pharmacological Aspects of Dopamine D4 Receptors. Nutrients.

[118] Cifani C., Koya E., Navarre B.M., Calu D.J., Baumann M.H., Marchant N.J., Liu Q.R., Khuc T., Pickel J., Lupica C.R. (2012). Medial prefrontal cortex neuronal activation and synaptic alterations after stress-induced reinstatement of palatable food seeking: A study using c-fos-GFP transgenic female rats. J. Neurosci..

[119] Zheng H., Corkern M.M., Crousillac S.M., Patterson L.M., Phifer C.B., Berthoud H.R. (2002). Neurochemical phenotype of hypothalamic neurons showing Fos expression 23 h after intracranial AgRP. Am. J. Physiol. Regul. Integr. Comp. Physiol..

[120] Stuber G.D., Wise R.A. (2016). Lateral hypothalamic circuits for feeding and reward. Nat. Neurosci..

[121] Piccoli L., Micioni Di Bonaventura M.V., Cifani C., Costantini V.J., Massagrande M., Montanari D., Martinelli P., Antolini M., Ciccocioppo R., Massi M. (2012). Role of orexin-1 receptor mechanisms on compulsive food consumption in a model of binge eating in female rats. Neuropsychopharmacology.

[122] Roseberry A.G. (2013). Altered feeding and body weight following melanocortin administration to the ventral tegmental area in adult rats. Psychopharmacology.

[123] Yen H.H., Roseberry A.G. (2015). Decreased consumption of rewarding sucrose solutions after injection of melanocortins into the ventral tegmental area of rats. Psychopharmacology.

[124] Shanmugarajah L., Dunigan A.I., Frantz K.J., Roseberry A.G. (2017). Altered sucrose self-administration following injection of melanocortin receptor agonists and antagonists into the ventral tegmental area. Psychopharmacology.

[125] Potoczna N., Branson R., Kral J.G., Piec G., Steffen R., Ricklin T., Hoehe M.R., Lentes K.U., Horber F.F. (2004). Gene variants and binge eating as predictors of comorbidity and outcome of treatment in severe obesity. J. Gastrointest. Surg..

[126] Sina M., Hinney A., Ziegler A., Neupert T., Mayer H., Siegfried W., Blum W.F., Remschmidt H., Hebebrand J. (1999). Phenotypes in three pedigrees with autosomal dominant obesity caused by haploinsufficiency mutations in the melanocortin-4 receptor gene. Am. J. Hum. Genet..

[127] Spitzer R.L., Yanovski S., Wadden T., Wing R., Marcus M.D., Stunkard A., Devlin M., Mitchell J., Hasin D., Horne R.L. (1993). Binge eating disorder: Its further validation in a multisite study. Int. J. Eat. Disord..

[128] Gotoda T. (2003). Binge eating as a phenotype of melanocortin 4 receptor gene mutations. N. Engl. J. Med..

[129] Hebebrand J., Geller F., Dempfle A., Heinzel-Gutenbrunner M., Raab M., Gerber G., Wermter A.K., Horro F.F., Blundell J., Schafer H. (2004). Binge-eating episodes are not characteristic of carriers of melanocortin-4 receptor gene mutations. Mol. Psychiatry.

[130] Lubrano-Berthelier C., Dubern B., Lacorte J.M., Picard F., Shapiro A., Zhang S., Bertrais S., Hercberg S., Basdevant A., Clement K. (2006). Melanocortin 4 receptor mutations in a large cohort of severely obese adults: Prevalence, functional classification, genotype-phenotype relationship, and lack of association with binge eating. J. Clin. Endocrinol. Metab..

[131] Tao Y.X., Segaloff D.L. (2005). Functional analyses of melanocortin-4 receptor mutations identified from patients with binge eating disorder and nonobese or obese subjects. J. Clin. Endocrinol. Metab..

[132] Gamero-Villarroel C., Rodriguez-Lopez R., Jimenez M., Carrillo J.A., Garcia-Herraiz A., Albuquerque D., Flores I., Gervasini G. (2015). Melanocortin-4 receptor gene variants are not associated with binge-eating behavior in nonobese patients with eating disorders. Psychiatr. Genet..

[133] Valette M., Poitou C., Le Beyec J., Bouillot J.L., Clement K., Czernichow S. (2012). Melanocortin-4 receptor mutations and polymorphisms do not affect weight loss after bariatric surgery. PLoS ONE.

[134] Bonnefond A., Keller R., Meyre D., Stutzmann F., Thuillier D., Stefanov D.G., Froguel P., Horber F.F., Kral J.G. (2016). Eating Behavior, Low-Frequency Functional Mutations in the Melanocortin-4 Receptor (MC4R) Gene, and Outcomes of Bariatric Operations: A 6-Year Prospective Study. Diabetes Care.

[135] Valette M., Poitou C., Kesse-Guyot E., Bellisle F., Carette C., Le Beyec J., Hercberg S., Clement K., Czernichow S. (2014). Association between melanocortin-4 receptor mutations and eating behaviors in obese patients: A case-control study. Int. J. Obes..

[136] Van der Klaauw A.A., von dem Hagen E.A., Keogh J.M., Henning E., O’Rahilly S., Lawrence A.D., Calder A.J., Farooqi I.S. (2014). Obesity-associated melanocortin-4 receptor mutations are associated with changes in the brain response to food cues. J. Clin. Endocrinol. Metab..

[137] Furlong T.M., Jayaweera H.K., Balleine B.W., Corbit L.H. (2014). Binge-like consumption of a palatable food accelerates habitual control of behavior and is dependent on activation of the dorsolateral striatum. J. Neurosci..

[138] Wang G.J., Geliebter A., Volkow N.D., Telang F.W., Logan J., Jayne M.C., Galanti K., Selig P.A., Han H., Zhu W. (2011). Enhanced striatal dopamine release during food stimulation in binge eating disorder. Obesity.

[139] Thorleifsson G., Walters G.B., Gudbjartsson D.F., Steinthorsdottir V., Sulem P., Helgadottir A., Styrkarsdottir U., Gretarsdottir S., Thorlacius S., Jonsdottir I. (2009). Genome-wide association yields new sequence variants at seven loci that associate with measures of obesity. Nat. Genet..

[140] Loos R.J., Lindgren C.M., Li S., Wheeler E., Zhao J.H., Prokopenko I., Inouye M., Freathy R.M., Attwood A.P., Beckmann J.S. (2008). Common variants near MC4R are associated with fat mass, weight and risk of obesity. Nat. Genet..

[141] Horstmann A., Kovacs P., Kabisch S., Boettcher Y., Schloegl H., Tonjes A., Stumvoll M., Pleger B., Villringer A. (2013). Common genetic variation near MC4R has a sex-specific impact on human brain structure and eating behavior. PLoS ONE.

[142] Qi L., Kraft P., Hunter D.J., Hu F.B. (2008). The common obesity variant near MC4R gene is associated with higher intakes of total energy and dietary fat, weight change and diabetes risk in women. Hum. Mol. Genet..

[143] Stutzmann F., Cauchi S., Durand E., Calvacanti-Proenca C., Pigeyre M., Hartikainen A.L., Sovio U., Tichet J., Marre M., Weill J. (2009). Common genetic variation near MC4R is associated with eating behaviour patterns in European populations. Int. J. Obes..

[144] Yilmaz Z., Davis C., Loxton N.J., Kaplan A.S., Levitan R.D., Carter J.C., Kennedy J.L. (2015). Association between MC4R rs17782313 polymorphism and overeating behaviors. Int. J. Obes..

[145] Blaine R.E., Kachurak A., Davison K.K., Klabunde R., Fisher J.O. (2017). Food parenting and child snacking: A systematic review. Int. J. Behav. Nutr. Phys. Act..

[146] Potter M., Vlassopoulos A., Lehmann U. (2018). Snacking Recommendations Worldwide: A Scoping Review. Adv. Nutr..

[147] Acosta A., Camilleri M., Shin A., Carlson P., Burton D., O’Neill J., Eckert D., Zinsmeister A.R. (2014). Association of melanocortin 4 receptor gene variation with satiation and gastric emptying in overweight and obese adults. Genes Nutr..

[148] Valladares M., Dominguez-Vasquez P., Obregon A.M., Weisstaub G., Burrows R., Maiz A., Santos J.L. (2010). Melanocortin-4 receptor gene variants in Chilean families: Association with childhood obesity and eating behavior. Nutr. Neurosci..

[149] Wardle J., Guthrie C.A., Sanderson S., Rapoport L. (2001). Development of the Children’s Eating Behaviour Questionnaire. J. Child Psychol. Psychiatry Allied Discip..

[150] De Lauzon B., Romon M., Deschamps V., Lafay L., Borys J.M., Karlsson J., Ducimetiere P., Charles M.A., Fleurbaix Laventie Ville Sante Study G. (2004). The Three-Factor Eating Questionnaire-R18 is able to distinguish among different eating patterns in a general population. J. Nutr..

[151] Turton R., Chami R., Treasure J. (2017). Emotional Eating, Binge Eating and Animal Models of Binge-Type Eating Disorders. Curr. Obes. Rep..

[152] Van Strien T., Cebolla A., Etchemendy E., Gutierrez-Maldonado J., Ferrer-Garcia M., Botella C., Banos R. (2013). Emotional eating and food intake after sadness and joy. Appetite.

[153] Obregon A.M., Oyarce K., Santos J.L., Valladares M., Goldfield G. (2017). Association of the melanocortin 4 receptor gene rs17782313 polymorphism with rewarding value of food and eating behavior in Chilean children. J. Physiol. Biochem..

[154] Yilmaz Z., Kaplan A.S., Tiwari A.K., Levitan R.D., Piran S., Bergen A.W., Kaye W.H., Hakonarson H., Wang K., Berrettini W.H. (2014). The role of leptin, melanocortin, and neurotrophin system genes on body weight in anorexia nervosa and bulimia nervosa. J. Psychiatr. Res..

[155] Abadi A., Peralta-Romero J., Suarez F., Gomez-Zamudio J., Burguete-Garcia A.I., Cruz M., Meyre D. (2016). Assessing the effects of 35 European-derived BMI-associated SNPs in Mexican children. Obesity.

[156] Cheung C.Y., Tso A.W., Cheung B.M., Xu A., Ong K.L., Fong C.H., Wat N.M., Janus E.D., Sham P.C., Lam K.S. (2010). Obesity susceptibility genetic variants identified from recent genome-wide association studies: Implications in a Chinese population. J. Clin. Endocrinol. Metab..

[157] El Hajj Chehadeh S., Osman W., Nazar S., Jerman L., Alghafri A., Sajwani A., Alawlaqi M., AlObeidli M., Jelinek H.F., AlAnouti F. (2020). Implication of genetic variants in overweight and obesity susceptibility among the young Arab population of the United Arab Emirates. Gene.

[158] Khalilitehrani A., Qorbani M., Hosseini S., Pishva H. (2015). The association of MC4R rs17782313 polymorphism with dietary intake in Iranian adults. Gene.

[159] Lv D., Zhang D.D., Wang H., Zhang Y., Liang L., Fu J.F., Xiong F., Liu G.L., Gong C.X., Luo F.H. (2015). Genetic variations in SEC16B, MC4R, MAP2K5 and KCTD15 were associated with childhood obesity and interacted with dietary behaviors in Chinese school-age population. Gene.

[160] Rana S., Rahmani S., Mirza S. (2018). MC4R variant rs17782313 and manifestation of obese phenotype in Pakistani females. RSC Adv..

[161] Shi J., Long J., Gao Y.T., Lu W., Cai Q., Wen W., Zheng Y., Yu K., Xiang Y.B., Hu F.B. (2010). Evaluation of genetic susceptibility loci for obesity in Chinese women. Am. J. Epidemiol..

[162] Zobel D.P., Andreasen C.H., Grarup N., Eiberg H., Sorensen T.I., Sandbaek A., Lauritzen T., Borch-Johnsen K., Jorgensen T., Pedersen O. (2009). Variants near MC4R are associated with obesity and influence obesity-related quantitative traits in a population of middle-aged people: Studies of 14,940 Danes. Diabetes.

[163] Orkunoglu-Suer F.E., Harmon B.T., Gordish-Dressman H., Clarkson P.M., Thompson P.D., Angelopoulos T.J., Gordon P.M., Hubal M.J., Moyna N.M., Pescatello L.S. (2011). MC4R variant is associated with BMI but not response to resistance training in young females. Obesity.

[164] Stevenson R.J., Francis H.M. (2017). The hippocampus and the regulation of human food intake. Psychol. Bull..

[165] Uher R., Murphy T., Brammer M.J., Dalgleish T., Phillips M.L., Ng V.W., Andrew C.M., Williams S.C., Campbell I.C., Treasure J. (2004). Medial prefrontal cortex activity associated with symptom provocation in eating disorders. Am. J. Psychiatry.

[166] Freeman L.M., Gil K.M. (2004). Daily stress, coping, and dietary restraint in binge eating. Int. J. Eat. Disord..

[167] Cifani C., Di Bonaventura M.V.M., Ciccocioppo R., Massi M. (2013). Binge eating in female rats induced by yo-yo dieting and stress. Animal Models of Eating Disorders.

[168] Polivy J., Zeitlin S.B., Herman C.P., Beal A.L. (1994). Food restriction and binge eating: A study of former prisoners of war. J. Abnorm. Psychol..

[169] Woods A.M., Racine S.E., Klump K.L. (2010). Examining the relationship between dietary restraint and binge eating: Differential effects of major and minor stressors. Eat. Behav..

[170] Gluck M.E., Geliebter A., Hung J., Yahav E. (2004). Cortisol, hunger, and desire to binge eat following a cold stress test in obese women with binge eating disorder. Psychosom. Med..

[171] Gluck M.E., Geliebter A., Lorence M. (2004). Cortisol stress response is positively correlated with central obesity in obese women with binge eating disorder (BED) before and after cognitive-behavioral treatment. Ann. N. Y. Acad. Sci..

[172] Cifani C., Micioni Di B.M., Vitale G., Ruggieri V., Ciccocioppo R., Massi M. (2010). Effect of salidroside, active principle of Rhodiola rosea extract, on binge eating. Physiol. Behav..

[173] Micioni Di Bonaventura M.V., Vitale G., Massi M., Cifani C. (2012). Effect of Hypericum perforatum Extract in an Experimental Model of Binge Eating in Female Rats. J. Obes..

[174] Epel E., Lapidus R., McEwen B., Brownell K. (2001). Stress may add bite to appetite in women: A laboratory study of stress-induced cortisol and eating behavior. Psychoneuroendocrinology.

[175] Coutinho W.F., Moreira R.O., Spagnol C., Appolinario J.C. (2007). Does binge eating disorder alter cortisol secretion in obese women?. Eat. Behav..

[176] Chaki S., Okubo T. (2007). Melanocortin-4 receptor antagonists for the treatment of depression and anxiety disorders. Curr. Top. Med. Chem..

[177] Chaki S., Okuyama S. (2005). Involvement of melanocortin-4 receptor in anxiety and depression. Peptides.

[178] Adan R.A., Szklarczyk A.W., Oosterom J., Brakkee J.H., Nijenhuis W.A., Schaaper W.M., Meloen R.H., Gispen W.H. (1999). Characterization of melanocortin receptor ligands on cloned brain melanocortin receptors and on grooming behavior in the rat. Eur. J. Pharmacol..

[179] De Barioglio S.R., Lezcano N., Celis M.E. (1991). Alpha MSH-induced excessive grooming behavior involves a GABAergic mechanism. Peptides.

[180] Spruijt B.M., van Hooff J.A., Gispen W.H. (1992). Ethology and neurobiology of grooming behavior. Physiol. Rev..

[181] Mul J.D., van Boxtel R., Bergen D.J., Brans M.A., Brakkee J.H., Toonen P.W., Garner K.M., Adan R.A., Cuppen E. (2012). Melanocortin receptor 4 deficiency affects body weight regulation, grooming behavior, and substrate preference in the rat. Obesity.

[182] Yamano Y., Yoshioka M., Toda Y., Oshida Y., Chaki S., Hamamoto K., Morishima I. (2004). Regulation of CRF, POMC and MC4R gene expression after electrical foot shock stress in the rat amygdala and hypothalamus. J. Vet. Med Sci..

[183] Davis M., Shi C. (1999). The extended amygdala: Are the central nucleus of the amygdala and the bed nucleus of the stria terminalis differentially involved in fear versus anxiety?. Ann. N. Y. Acad. Sci..

[184] Blasio A., Iemolo A., Sabino V., Petrosino S., Steardo L., Rice K.C., Orlando P., Iannotti F.A., Di Marzo V., Zorrilla E.P. (2013). Rimonabant precipitates anxiety in rats withdrawn from palatable food: Role of the central amygdala. Neuropsychopharmacology.

[185] Micioni Di Bonaventura M.V., Pucci M., Giusepponi M.E., Romano A., Lambertucci C., Volpini R., Micioni Di Bonaventura E., Gaetani S., Maccarrone M., D’Addario C. (2019). Regulation of adenosine A2A receptor gene expression in a model of binge eating in the amygdaloid complex of female rats. J. Psychopharmacol..

[186] Karami Kheirabad M., Namavar Jahromi B., Tamadon A., Ramezani A., Ahmadloo S., Sabet Sarvestan F., Koohi-Hosseinabadi O. (2015). Expression of Melanocortin-4 Receptor mRNA in Male Rat Hypothalamus During Chronic Stress. Int. J. Mol. Cell. Med..

[187] Chagra S.L., Zavala J.K., Hall M.V., Gosselink K.L. (2011). Acute and repeated restraint differentially activate orexigenic pathways in the rat hypothalamus. Regul. Pept..

[188] Von Frijtag J.C., Croiset G., Gispen W.H., Adan R.A., Wiegant V.M. (1998). The role of central melanocortin receptors in the activation of the hypothalamus-pituitary-adrenal-axis and the induction of excessive grooming. Br. J. Pharmacol..

[189] Lu X.Y., Barsh G.S., Akil H., Watson S.J. (2003). Interaction between alpha-melanocyte-stimulating hormone and corticotropin-releasing hormone in the regulation of feeding and hypothalamo-pituitary-adrenal responses. J. Neurosci..

[190] Hwang B.H., Guntz J.M. (1997). Downregulation of corticotropin-releasing factor mRNA, but not vasopressin mRNA, in the paraventricular hypothalamic nucleus of rats following nutritional stress. Brain Res. Bull..

[191] Liposits Z., Sievers L., Paull W.K. (1988). Neuropeptide-Y and ACTH-immunoreactive innervation of corticotropin releasing factor (CRF)-synthesizing neurons in the hypothalamus of the rat. An immunocytochemical analysis at the light and electron microscopic levels. Histochemistry.

[192] Sarkar S., Legradi G., Lechan R.M. (2002). Intracerebroventricular administration of alpha-melanocyte stimulating hormone increases phosphorylation of CREB in TRH- and CRH-producing neurons of the hypothalamic paraventricular nucleus. Brain Res..

[193] Liu J., Garza J.C., Truong H.V., Henschel J., Zhang W., Lu X.Y. (2007). The melanocortinergic pathway is rapidly recruited by emotional stress and contributes to stress-induced anorexia and anxiety-like behavior. Endocrinology.

[194] Ulrich-Lai Y.M., Herman J.P. (2009). Neural regulation of endocrine and autonomic stress responses. Nat. Rev. Neurosci..

[195] Bedse G., Romano A., Tempesta B., Lavecchia M.A., Pace L., Bellomo A., Duranti A., Micioni Di Bonaventura M.V., Cifani C., Cassano T. (2015). Inhibition of anandamide hydrolysis enhances noradrenergic and GABAergic transmission in the prefrontal cortex and basolateral amygdala of rats subjected to acute swim stress. J. Neurosci. Res..

[196] Dayas C.V., Buller K.M., Day T.A. (1999). Neuroendocrine responses to an emotional stressor: Evidence for involvement of the medial but not the central amygdala. Eur. J. Neurosci..

[197] Roozendaal B., McEwen B.S., Chattarji S. (2009). Stress, memory and the amygdala. Nat. Rev. Neurosci..

[198] Choi D.C., Furay A.R., Evanson N.K., Ostrander M.M., Ulrich-Lai Y.M., Herman J.P. (2007). Bed nucleus of the stria terminalis subregions differentially regulate hypothalamic-pituitary-adrenal axis activity: Implications for the integration of limbic inputs. J. Neurosci..

[199] Forray M.I., Gysling K. (2004). Role of noradrenergic projections to the bed nucleus of the stria terminalis in the regulation of the hypothalamic-pituitary-adrenal axis. Brain Res. Brain Res. Rev..

[200] Micioni Di Bonaventura M.V., Ciccocioppo R., Romano A., Bossert J.M., Rice K.C., Ubaldi M., St Laurent R., Gaetani S., Massi M., Shaham Y. (2014). Role of bed nucleus of the stria terminalis corticotrophin-releasing factor receptors in frustration stress-induced binge-like palatable food consumption in female rats with a history of food restriction. J. Neurosci..

[201] Micioni Di Bonaventura M.V., Ubaldi M., Giusepponi M.E., Rice K.C., Massi M., Ciccocioppo R., Cifani C. (2017). Hypothalamic CRF1 receptor mechanisms are not sufficient to account for binge-like palatable food consumption in female rats. Int. J. Eat. Disord..

[202] Chaffin A.T., Fang Y., Larson K.R., Mul J.D., Ryan K.K. (2019). Sex-dependent effects of MC4R genotype on HPA axis tone: Implications for stress-associated cardiometabolic disease. Stress.

[203] Ryan K.K., Mul J.D., Clemmensen C., Egan A.E., Begg D.P., Halcomb K., Seeley R.J., Herman J.P., Ulrich-Lai Y.M. (2014). Loss of melanocortin-4 receptor function attenuates HPA responses to psychological stress. Psychoneuroendocrinology.

[204] Cottone P., Sabino V., Roberto M., Bajo M., Pockros L., Frihauf J.B., Fekete E.M., Steardo L., Rice K.C., Grigoriadis D.E. (2009). CRF system recruitment mediates dark side of compulsive eating. Proc. Natl. Acad. Sci. USA.

[205] Iemolo A., Blasio A., St Cyr S.A., Jiang F., Rice K.C., Sabino V., Cottone P. (2013). CRF-CRF1 receptor system in the central and basolateral nuclei of the amygdala differentially mediates excessive eating of palatable food. Neuropsychopharmacology.

[206] Romano A., Micioni Di Bonaventura M.V., Gallelli C.A., Koczwara J.B., Smeets D., Giusepponi M.E., De Ceglia M., Friuli M., Micioni Di Bonaventura E., Scuderi C. (2020). Oleoylethanolamide decreases frustration stress-induced binge-like eating in female rats: A novel potential treatment for binge eating disorder. Neuropsychopharmacology.

[207] Cifani C., Micioni Di Bonaventura E., Botticelli L., Del Bello F., Giorgioni G., Pavletic P., Piergentili A., Quaglia W., Bonifazi A., Schepmann D. (2020). Novel Highly Potent and Selective Sigma1 Receptor Antagonists Effectively Block the Binge Eating Episode in Female Rats. ACS Chem. Neurosci..

[208] Pucci M., Micioni Di Bonaventura M.V., Zaplatic E., Bellia F., Maccarrone M., Cifani C., D’Addario C. (2018). Transcriptional regulation of the endocannabinoid system in a rat model of binge-eating behavior reveals a selective modulation of the hypothalamic fatty acid amide hydrolase gene. Int. J. Eat. Disord..

[209] Micioni Di Bonaventura M.V., Cifani C., Lambertucci C., Volpini R., Cristalli G., Massi M. (2012). A2A adenosine receptor agonists reduce both high-palatability and low-palatability food intake in female rats. Behav. Pharmacol..

